# A comparative analysis of protein targets of withdrawn cardiovascular drugs in human and mouse

**DOI:** 10.1186/2043-9113-2-10

**Published:** 2012-05-01

**Authors:** Yuqi Zhao, Jingwen Wang, Yanjie Wang, Jingfei Huang

**Affiliations:** 1 State Key Laboratory of Genetic Resources and Evolution, Kunming Institute of Zoology, Chinese Academy of Sciences, 32, Eastern Jiaochang Road, Kunming, Yunnan, 650223, China; 2 Yantai Yuhuangding Hospital, Yantai, Shandong Province, 264000, China; 3 Key Laboratory of Animal Models and Human Disease Mechanisms of Chinese Academy of Sciences and Yunnan Province, Kunming Institute of Zoology, Chinese Academy of Sciences, Kunming, Yunnan, 650223, China; 4 Kunming Institute of Zoology-Chinese University of Hongkong Joint Research Center for Bio-resources and Human Disease Mechanisms, Kunming, 650223, China

**Keywords:** Withdrawn cardiovascular drugs, Animal modeling, Sequence divergence, Side effects, Drug-binding pocket

## Abstract

**Background:**

Mouse is widely used in animal testing of cardiovascular disease. However, a large number of cardiovascular drugs that have been experimentally proved to work well on mouse were withdrawn because they caused adverse side effects in human.

**Methods:**

In this study, we investigate whether binding patterns of withdrawn cardiovascular drugs are conserved between mouse and human through computational dockings and molecular dynamic simulations. In addition, we also measured the level of conservation of gene expression patterns of the drug targets and their interacting partners by analyzing the microarray data.

**Results:**

The results show that target proteins of withdrawn cardiovascular drugs are functionally conserved between human and mouse. However, all the binding patterns of withdrawn drugs we retrieved show striking difference due to sequence divergence in drug-binding pocket, mainly through loss or gain of hydrogen bond donors and distinct drug-binding pockets. The binding affinities of withdrawn drugs to their receptors tend to be reduced from mouse to human. In contrast, the FDA-approved and best-selling drugs are little affected.

**Conclusions:**

Our analysis suggests that sequence divergence in drug-binding pocket may be a reasonable explanation for the discrepancy of drug effects between animal models and human.

## Introduction

Mouse is the most commonly used vertebrate species in animal testing which is a vital part of drug development. Currently, all new pharmaceuticals undergo rigorous animal testing before being licensed for human use. It is commonly accepted that animal testing on mouse can provide us critical information for assessing hazard and risk potential [1,2] . However, many withdrawn pharmaceuticals worked very well in animal models while led to adverse side effects or failed to reach the expected effects in human. Over sixty drugs ever approved by the FDA were withdrawn in the past twenty years [3]. A recent and well-known example is the failure of an Alzheimer’s drug Dimebon (latrepirdine). The drug improved the performance of memory-impaired mice and rats [4,5], but failed to show a significant effect in the phase III clinical trial. Inevitably, a question arises as to why the discrepancy of drug effects between animal models and human. Answering this question will help us avoid drug withdrawals as much as possible and ‘rescue’ some of the withdrawn drugs by promoting genotype-based prescribing.

We presumed that two aspects would be the prime determinants of the discrepancy of drug response between animal models and humans. First of all, target proteins of withdrawn drugs may be functionally divergent between human and mouse. Phenotypic differences between species might be caused by changes in species-specific interactions or gene expression [6-8], which also lead to the differential responses to the exogenous drugs. Secondly, three-dimensional structures of drug targets may have changed due to sequence divergence in the drug-binding pockets. As a result, although the drug targets are functionally conserved, the binding patterns of drugs with these targets may change from animal models to human.

In this study, both genetic aspects were examined to elucidate the mechanism of discrepancy of withdrawn drugs between human and mouse. We focused on withdrawn cardiovascular drugs, not only because cardiovascular drugs are in a position of importance in all pharmaceuticals, but also because the relevant information is comprehensive, including target, pharmacodynamics and so on. The results show that the drug targets are not involved in species-specific interactions. The binding patterns of withdrawn cardiovascular drugs are indeed affected by sequence divergence in the drug-binding pockets. The trend of binding affinity of these withdrawn drugs is to be reduced from mouse to human. Our study gleans valuable insights into the development of cardiovascular drugs and may reduce the amount of laboratory effort required in this process.

## Results

### Target proteins of withdrawn cardiovascular drugs are functionally conserved between human and mouse

In order to compare the target protein of withdrawn cardiovascular drugs between human and mouse, we first examined the orthologous relationships of these proteins through Ensembl gene orthology prediction pipeline (http://www.ensembl.org/). It shows that all of the ortholog relationships (25/25) are one to one (Additional file 1: Table S1), which are widely assumed to have similar functions and cause similar phenotypes [9].

**Figure 1:**
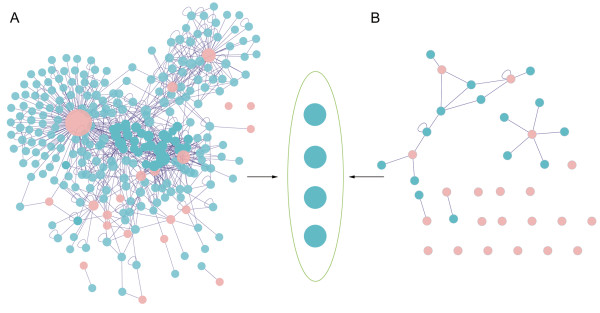
**Protein interaction networks of targets of withdrawn cardiovascular drugs.** The interaction networks of human (panel **A**) and mouse (panel **B**) drug targets are displayed respectively. The pink nodes represent the drug targets, and the blue ones are proteins that have been experimentally proved to interact with drug targets. The edges between nodes represent interactions. The ellipse represents overlap between two networks. There are only four nodes to be found.

We further detected whether targets of withdrawn cardiovascular drugs were involved in species-specific interactions by reconstructing interaction networks for human and mouse drug targets separately through integrating experimental datasets (Materials and methods). To our surprise, there is only a narrow overlap between the reconstructed human and mouse drug target interaction networks (Figure 1). Besides the drug targets, only four proteins (Nomenclature gene names are CCP110, HDAC3, KCNMA1 and PPARGC1A) are found in both networks. We supposed that it was mainly caused by inadequacy of information in databases and confirmed each interaction by computational methods, such as literature search, gene co-expression and bayesian networks (Additional file 1: Table S2) [10]. The results indicate that most of the interactions related to withdrawn cardiovascular drug targets are highly preserved between human and mouse.

In addition, we reasoned that changes in gene expression underlie many of the phenotypic differences between species and lead to differential responses to withdrawn drugs. As a result, we also measured the level of conservation of gene expression patterns of the drug targets and their interacting partners (Materials and methods). For the expression profiles of these orthologous genes between mouse and human, are the average Pearson’s correlation coefficient r is 0.27 and is significantly higher than that for the random gene pairs (Student’s two sample *t*-test; *p* <10^−8^). It shows that the gene expression evolution of these drug targets between human and mouse was strongly shaped by purifying selection, suggesting that gene expression variation may not have an influence on the drug effect.

### Sequence divergence near the functional sites

We reasoned that non-conserved substitutions of critical residues in drug targets could lead to different response to drugs. As a result, whether these residues had changed from mouse to human was analyzed. The results shows that the critical sites of drug targets, such as active sites and phosphorylated residues, are conserved while residues nearby have changed a lot from mouse to human, including losses of hydrogen bond donors and substitutions of amino acid residues of opposite charge (Additional file 1: SI Table S1). For example, human plasminogen activator inhibitor 1, the target of troglitazone (Drug Bank ID: DB00197), shares only 78% sequence identity with its mouse ortholog. The critical sites are Arg369 and Met370 [11], which are conserved, while the residues within 10 Å distance in three-dimensional structure have changed a lot, such as Lys311Gly, His339Ser, and Glu373Thr. Previous work revealed that drug-binding sites on proteins usually exist out of functional necessity [12], so we can infer that the binding pockets of withdrawn drugs could be affected by sequence divergence.

### Structural modeling of mouse drug targets and refinement by molecular dynamics simulation

**Figure 2:**
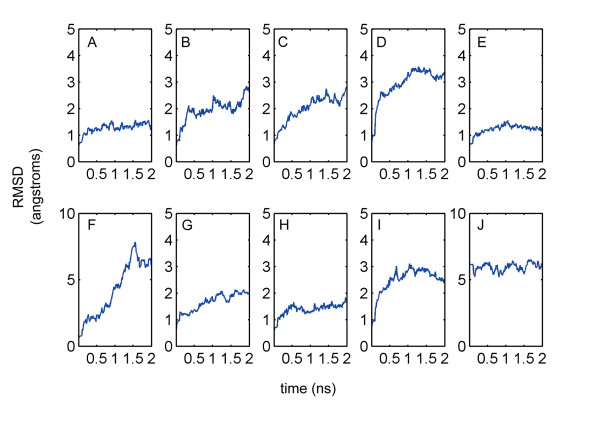
**RMSD values of mutant human protein structures during the dynamics simulations.** The figures **A-J** respectively correspond to mutants of crystal structures (PDB code 1C5G, 1DQA, 1HWL, 1Y9C, 2BVR, 2KBI, 2QT9, 3D24, 3DZY and 3G43).

Because the structures of mouse drug targets are unsolved, we generated mutant models of human target proteins as rational mouse three-dimensional structures and employed molecular dynamics simulations for structural refinement. According to previous work that MD simulations on the nanosecond time scale were sufficient for refinement of protein models [13], simulations for 2 nanoseconds were first performed. The stability of each simulation was monitored through examination of structural properties which occurred during the course of the 2 nanoseconds production trajectories. The plots of root-mean-square-deviation (RMSD) from the original starting coordinates indicate that seven models are well-behaved which means the simulations are reasonably converged and protein structures are well refined (Figure 2 A, C-E, G-I). Interestingly, a relatively large shift in RMSD (Figure 2F) at around 0.5 ns suggests that this structure is not well refined. It may be caused by the large loop (residues 1769–1787). The simulations of other two structures (Figure 2B, J) are also not well converged. As a result, we further performed simulations of these two structures for 5 nanoseconds until the RMSD values show only minor variation.

The root mean-square fluctuations (RMSF) of the mutated residues in ten drug targets were also computed to examine the impact of mutating these residues on conformational changes. The average RMSF of the mutated residues is significant higher than that of the conserved residues in 2 ns (Student’s two-sample *t*-test, *p* = 3.52 × 10^−7^), indicating that mutated residues have a great influence on conformational changes of drug targets.

### Differences in the binding patterns of withdrawn cardiovascular drugs to their targets between human and mouse

We next analyzed whether the drugs might bind human and mouse drug targets with different patterns and affinities. These drugs belong to different categories (Additional file 1: Table S1) and so are demonstrated separately.

**Figure 3:**
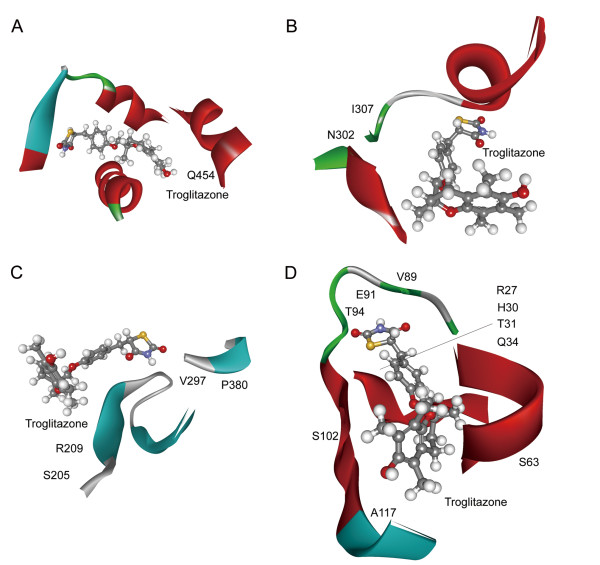
**Binding patterns of troglitazone with its targets.** Panel (**A**) illustrates interactions between troglitazone and human PPAR-gamma, while (**B**) represents interactions between drug and corresponding mouse target. Panel (**C**) illustrates interactions between troglitazone and human PAI, while (**D**) represents interactions between drug and corresponding mouse target. The residue substitutions in the binding pocket are labeled.

Troglitazone works as vasodilator agents and platelet aggregation inhibitors [14]. It selectively target six proteins, of which four have crystal structures (Nomenclature gene names: ESRRA, ESRRG, SERPINE1 and PPARG). The interaction between troglitazone and Estrogen-related receptor gamma (ERR gamma-2) shows no interspecies difference because the protein sequences in human and mouse are identical. However, the other three targets that are not paralogs and perform different functions show variance in the drug-binding pockets. A few important amino acids within the binding pocket in human Peroxisome proliferator-activated receptor gamma (PPAR-gamma) have changed from mouse to human (Asn302Ser, Asn355Ser and His454Gln). It indicates that the troglitazone-binding pocket in mouse PPAR-gamma is distinct from that in human ortholog (Figure 3, A-B). The binding free energies demonstrate that the binding affinity of PPAR-gamma for troglitazone from mouse to human is reduced (Additional file 1: Table S3 and Materials and methods; Student’s two-sample *t*-test, *p* = 8.3 × 10^−5^). As to plasminogen activator inhibitor 1 (PAI), it shows that the residues in the binding pocket have changed greatly including, inter alia, Arg27Pro, His30Tyr, Thr31Val, Ser63Ala and so on (Figure 3, C-D). The binding energies also show that the specificity of interaction between troglitazone and receptor PAI is significantly different (Student’s two-sample *t*-test, *p* = 1.14 × 10^−10^). Unlike interacting with other targets, troglitazone binds to Steroid hormone receptor ERR1 as an inverse agonist. [15] There are only three amino acid substitutions of similar properties near the pocket (Additional file 1: Figure S1, A-B), and the receptor binding affinity is little affected (Student’s two-sample *t*-test, *p* = 0.078).

**Figure 4:**
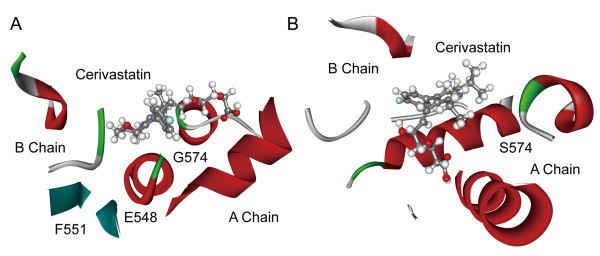
**Binding patterns of cerivastatin with its targets.** Panel (**A**) illustrates interactions between cerivastatin and human HMG-CoA reductase, while (**B**) represents interactions between drug and corresponding mouse target. The residue substitutions in the binding pocket and chains are labeled.

Cerivastatin acts as antagonist on HMG-CoA reductase, which functions as a homodimer and is the rate-limiting enzyme of sterol biosynthesis [16,17]. The computational docking results indicate that cerivastatin binds to the homodimer interface to keep it from dimerizing. The analysis shows that several critical residue substitutions (A: Tyr551Phe, A: Glu548Gly and A: Ser574Gly), which lead to the loss of hydrogen bond donors, account for the difference in the binding pattern of cerivastatin between human and mouse (Figure 4, A-B). The analysis of binding free energy also indicates that the binding affinity is reduced from mouse to human (Student’s two-sample *t*-test, *p* = 0.033).

Encainide can mediate the voltage-dependent sodium ion permeability of excitable membrane by blocking sodium channel protein type 5 subunit alpha (HH1) [18]. Two residue substitutions (Leu1812Ser and Ala1813Val) occurred in the Encainide binding pocket (Additional file 1: Figure S2, A-B). The amino acid residue Ser 1812 acts as a hydrogen donor according to the identification result of drug-binding sites. However, the mouse and human HH1 show similar encainide binding affinity. (Student’s two-sample *t*-test, *p* = 0.64).

**Figure 5:**
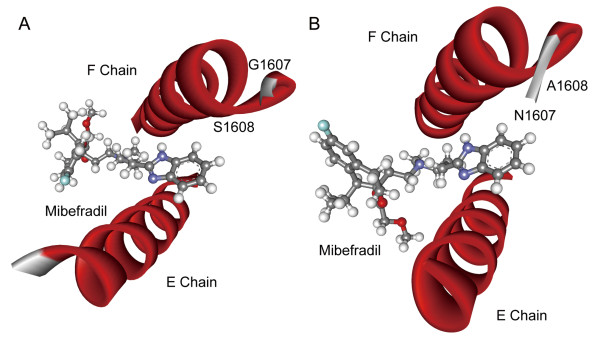
**Binding patterns of mibefradil with its targets.** Panel (**A**) illustrates interactions between mibefradil and human calcium channel, while (**B**) represents interactions between drug and corresponding mouse target. The residue substitutions in the binding pocket and chains are labeled.

Mibefradil belongs to calcium channel blockers. Its targets include T-type and L-type calcium channels, both of which mediate the entry of calcium ions into excitable cells and are also involved in a variety of calcium-dependent processes, such as muscle contraction, hormone or neurotransmitter release and so on [19,20]. Of the 11 mibefradil targets, only four proteins have peptide fragments resolved and one meets our standards (PDB ID code 3 G43). The computational docking results show that the binding pocket is composed of two helixes separately from two chains, in which two different amino acids (Asn1607Gly, Ala1608Ser) between species change the helix-helix interaction and so change the mibefradil binding pattern (Figure 5, A-B). The binding energy calculation also reveals that the binding affinity is reduced from mouse to human (Student’s two-sample *t*-test, *p* = 1.53 × 10^-4^).

**Figure 6:**
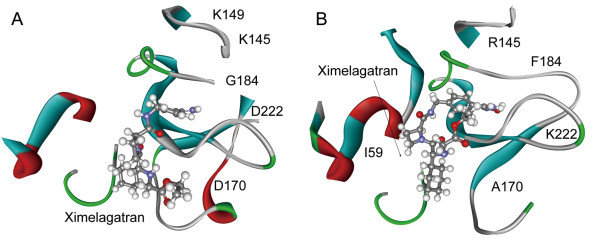
**Binding patterns of ximelagatran with its targets.** Panel (**A**) illustrates interactions between ximelagatran and human prothrombin, while (**B**) represents interactions between drug and corresponding mouse target. The residue substitutions in the binding pocket are labeled.

Ximelagatran was investigated as an anticoagulant, which inhibited prothrombin from converting fibrinogen to fibrin and activates factors [21]. The sequence identity of prothrombin between mouse and human was just 0.81, hinting that the drug-receptor interaction might also have interspecies specificity. Our results demonstrated that the residue substitutions (Glu149lys, Ala170Asp, Phe184Tyr, Lys222Asp and so on) in the binding pocket (Figure 6, A-B) changed the drug binding pattern. The binding affinity of ximelagatran was also affected between species (Student’s two-sample *t*-test, *p* = 3.8 × 10^−3^).

### The binding pattern and binding affinity is similar for FDA-approved drugs

We next inspected that whether sequence divergence affects the binding pattern and binding affinity of FDA-approved drugs. In comparison, the FDA-approved and best-selling drugs, atorvastatin and clopidogrel (brand names are lipitor and plavix separately) were estimated. The results show that the binding patterns and binding affinities of both drugs are little affected by sequence divergence between human and mouse (SI Text Results).

## Discussion

In this study, we demonstrate a reasonable possibility of the discrepancy of withdrawn cardiovascular drugs between mouse model and human. The drug targets are proved to be not involved in species-specific interactions. Then, reasonable models for mouse protein structures are generated through molecular dynamics simulations. After that, we determine the differences of binding patterns and affinities of the withdrawn cardiovascular drugs between human and mouse and two FDA-approved and best-selling drugs are selected as controls. Our results show that the binding patterns of withdrawn cardiovascular drugs in our study are indeed affected by sequence divergence, especially the non-conserved residue substitutions, such as Lys into Glu, Ser into Ala and so on. The trend of binding affinity of these withdrawn drugs is to be reduced from mouse to human, which may explain the low specificity of human targets. Finally, we explore whether off-target effects could be caused. It shows that more off-target effects can occur in human than in mouse due to the local structural similarities of drug-binding sites.

**Figure 7:**
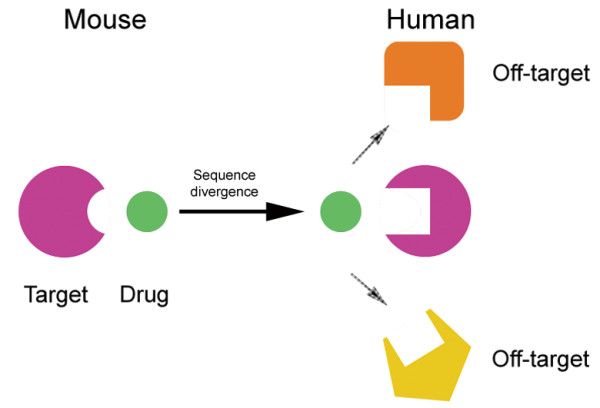
**Illustration of side-effects caused by sequence divergence.** Adverse side-effects may arise due to significant sequence divergence in the drug-binding pockets between mouse and human.

Usually, adverse side effects occur when the drug interacts with unintended targets [22]. Since the binding sites and affinity of withdrawn cardiovascular drugs were estimated to have changed from mouse to human, these drugs might interact with more off-targets in human than in mouse, due to similar local structures of drug-binding sites. So, we determined the cardiovascular drug-binding sites of human and mouse targets separately and then searched the similar local structures in structure database. It demonstrates that human targets of withdrawn drugs get 7 off-targets in all while mouse targets get no off-targets due to the differences of drug-binding sites (Materials and methods; Additional file 1: Table S4). Adverse side-effects may arise due to significant sequence divergence between mouse and human (Figure 7). Before drug testing, the animal model should be selected in a careful and meticulous manner, not only in CAD systems but also in other complex disease systems.

A key issue in translational medicine is the need to devote more attention to the characteristics of the drug–target interaction [23], yet most of target proteins in animal models have not or fragmentally solved. Besides, according to previous works, one missense mutation is sufficient to lead to altered binding pattern of drug targets [24,25]. Studies on structural divergence of drug targets between disease models and human provide valuable information for the development of human disease models. In this study, we employed MD simulations to construct models of good quality as target structures and then applied them to comparison of drug-target interactions. It will be very useful to identify a suitable model for animal testing, in case of false positive results supported by inappropriate experiments.

Although we highlight the contributions of sequence divergence, we also illustrate specific interactions that were experimentally confirmed in just one species. Recent studies have shown that species-specific interactions and regulations exist in human and mouse [26,27]. These species-specific properties may lead to different responses to the same pharmaceutical. Besides, false positive results in mouse may also lead to the success of animal testing while failure of clinical treatments. For example, prenylamine is a calcium channel blocker of the amphetamine chemical class which is used as a vasodilator in the treatment of angina pectoris. It has been shown to partially metabolize to amphetamine and can cause false positives for it in drug tests [28-30].

In summary, our results provide a reasonable explanation for the discrepancy of cardiovascular drugs. We will further testify for other pharmaceuticals.

## Materials and methods

### Collection of withdrawn and FDA-approved cardiovascular drugs

The information of withdrawn drug for CADs were retrieved from DrugBank database [31] and previous works [3]. Seven withdrawn cardiovascular drugs (Drug Bank ID: DB00197, DB00439, DB01228, DB01388, DB04825, DB04831 and DB04898) were included. In this dataset, prenylamine (Drug Bank ID: DB04825) was withdrawn for it partially metabolized to amphetamine and could cause false positives for it in drug tests [29]. It was just a prodrug, so it was excluded out of our study. Ticrynafen (Drug Bank ID: DB04831) was also excluded because it had no target information. We also chose two FDA-approved and the most sales-potential drugs, Lipitor (Drug Bank ID: DB01076) and Plavix (Drug Bank ID: DB00758) as positive control because the higher sales-potential might indicate the more satisfactory efficacy and less side-effect.

### Reconstruction of interaction networks of withdrawn drug targets and literature search

The interaction data was retrieved from several common databases, including DIP [32], BIND [33], HPRD [34], BioGRID [35], MINT [36] and IntAct [37]. A Cytoscape [38] plugin, BisoGenet, was applied to reconstruct interaction networks of human and mouse drug targets separately. Only the interactions that were verified by experiments were displayed. Another cytoscape plugin, Agilent Literature Search 2.71, was used to do literature search. Gene aliases and context were set to restrain the search results.

### Analysis of gene expression data

The microarray datasets (GSE8000 for mouse [39] and GSE11560 for human [40]) were downloaded from the Gene Expression Omnibus (GEO) (http://www.ncbi.nlm.nih.gov/geo). The expression profile of the drug targets were analyzed according to our previous work [41].

### Preparation of structures of drug targets

All the protein structure files of withdrawn drug targets were downloaded from PDB database [42]. Structures, containing the main functional domains and of which sequence coverage is above 80%, were adopted to do the subsequent analysis. If the sequence coverage was below the cutoff value and yet the drug-binding regions were intact and clearly reported in a structure, then the structure was also included in the dataset (Additional file 1: Table S1).

### Generation of mutant models for mouse targets and refinement

Mutant models of human protein structures were made as mouse protein structures using MODELER program. [43] High levels of molecular dynamics with simulated annealing were performed to optimize the local structures of mutant models. After that, molecular dynamics simulations were performed using NAMD and CHARMM31 force filed with CMAP correction [44,45]. The protein models were solvated in a TIP3P water box [46] and ionized by NaCl (0.152 M) to mimic physiological conditions. The ionized systems were minimized for 50,000 integration steps and equilibrated for 2 ns with 2 fs time stepping. Following this, a 2 ns unconstrained equilibration was performed for subsequent trajectory analysis, with frames stored each picosecond. Constant temperature (T = 310 K) was enforced using Langevin dynamics with a damping coefficient of 5 ps. Constant pressure (*p* = 1 atm) was enforced through the Nosé-Hoover Langevin piston method with a decay period of 100 fs and a damping time constant of 5 per picosecond. Van der Waals interaction cutoff distances were set at 12 Å, (smooth switching function beginning at 10 Å) and long-range electrostatic forces were computed using the particle-mesh Ewald (PME) with a grid size of less than 1.0 Å.

### Comparison of binding patterns of cardiovascular drugs

We compared modes of actions of withdrawn cardiovascular drugs following the steps below. Step one; we detect the drug binding pockets through a CHARMm-based molecular dynamics scheme [47]. We performed simulated annealing refinement and saved the top ten poses to prepare for the calculation of binding energy. Step two: a flexible docking method was applied to find the optimal binding sites by simulating protein flexibility and docking drugs with an induced fit receptor optimization [48]. Step three; we determined the critical drug-binding sites by using Receptor-Ligand Interactions tool. We set 2.3 Å as cutoff value to exclude the minor important bonds. All simulations reported above were carried out using Discovery Studio© v2.5.0.9164, built on the SciTegic Enterprise Server platform (Accelrys Inc, 2009).

### Detection of local structural similarities of drug-binding sites in structure databases

An accurate algorithm for detecting local protein structural similarity, CMASA [49] was applied to detect local structural similarities of drug-binding sites in non-redundant structure database from SCOP database [50]. The binding sites detected above were used as queries. Substitution files were applied in the process and the cutoff was set to the default value.

### Binding free energy calculations and statistical analysis

The drug-target binding free energies were calculated in the protocols of Discovery Studio, according to the previous work [51]. The values of the binding free energy (ΔG binding) for each drug-target complex were calculated based on the following equation:

(1) ΔG binding=ΔG complex –ΔG drug –ΔG target

We used the top ten poses mentioned above to calculate binding energies. In situ ligand minimization and ligand conformational entropy were set to default value. The distance cutoff value of ligand conformational entropy was set to 14.0 Å. If the ten calculated binding energies (Additional file 1: Table S3) followed a normal distribution, Student’s two-sample *t* test was applied to compare the binding affinity of drugs to their targets between human and mouse.

## Competing interests

The authors declare no competing financial interests.

## Author contributions

Yuqi Zhao conceived, designed and performed the experiments. Jingwen Wang and Yanjie Wang analyzed the results and revised the manuscript. Jingfei Huang supervised the work and wrote the manuscript with support from all authors. All authors read and approved the final manuscript.

## Supplementary Material

Additional file 1 Supporting Information. Click here for file

## References

[B1] Baker M Animal models: inside the minds of mice and men Nature 2011 475 123 128 10.1038/475123a 21734709

[B2] Dolgin E Animal testing alternatives come alive in US Nat Med 2010 16 1348 1348 10.1038/nm1210-1348 21135821

[B3] Shah RR Can pharmacogenetics help rescue drugs withdrawn from the market? Pharmacogenomics 2006 7 889 908 10.2217/14622416.7.6.889 16981848

[B4] Miller G PHARMACOLOGY The Puzzling Rise and Fall of a Dark-Horse Alzheimer’s Drug Science 2010 327 1309 1309 10.1126/science.327.5971.1309 20223954

[B5] Wang J Ferruzzi MG Varghese M Qian XJ Cheng A Xie M Zhao W Ho L Pasinetti GM Preclinical study of dimebon on beta-amyloid-mediated neuropathology in Alzheimer’s disease Molecular Neurodegeneration 2011 6 7 10.1186/1750-1326-6-7 21247479 PMC3035024

[B6] Benfey PN Mitchell-Olds T Perspective - From genotype to phenotype: Systems biology meets natural variation Science 2008 320 495 497 10.1126/science.1153716 18436781 PMC2727942

[B7] Miller JA Horvath S Geschwind DH Divergence of human and mouse brain transcriptome highlights Alzheimer disease pathways Proceedings of the National Academy of Sciences of the United States of America 2010 107 12698 12703 10.1073/pnas.0914257107 20616000 PMC2906579

[B8] Brawand D Soumillon M Necsulea A Julien P Csardi G Harrigan P Weier M Liechti A Aximu-Petri A Kircher M The evolution of gene expression levels in mammalian organs Nature 2011 478 343-+ 22012392 10.1038/nature10532

[B9] Liao BY Zhang JZ Null mutations in human and mouse orthologs frequently result in different phenotypes Proceedings of the National Academy of Sciences of the United States of America 2008 105 6987 6992 10.1073/pnas.0800387105 18458337 PMC2383943

[B10] Shoemaker BA Panchenko AR Deciphering protein-protein interactions. Part II. Computational methods to predict protein and domain interaction partners Plos Comput Biol 2007 3 595 601 10.1371/journal.pcbi.0030043 PMC1857810 17465672

[B11] Andreasen PA Riccio A Welinder KG Douglas R Sartorio R Nielsen LS Oppenheimer C Blasi F Dano K Plasminogen-Activator Inhibitor Type-1 - Reactive Center and Amino-Terminal Heterogeneity Determined by Protein and Cdna Sequencing Febs Lett 1986 209 213 218 10.1016/0014-5793(86)81113-9 3025016

[B12] Hopkins AL Groom CR The druggable genome Nat Rev Drug Discov 2002 1 727 730 10.1038/nrd892 12209152

[B13] Fan H Mark AE Refinement of homology-based protein structures by molecular dynamics simulation techniques Protein Sci 2004 13 211 220 10.1110/ps.03381404 14691236 PMC2286528

[B14] Aljada A Garg R Ghanim H Mohanty P Hamouda W Assian E Dandona P Nuclear factor-kappa B suppressive and inhibitor-kappa B stimulatory effects of troglitazone in obese patients with type 2 diabetes: Evidence of an antiinflammatory action? J Clin Endocr Metab 2001 86 3250 3256 10.1210/jc.86.7.3250 11443197

[B15] Wang Y Fang F Wong CW Troglitazone is an estrogen-related receptor alpha and gamma inverse agonist Biochem Pharmacol 2010 80 80 85 10.1016/j.bcp.2010.03.013 20298676

[B16] Ganne F Vasse M Beaudeux JL Peynet J Francois A Mishal Z Chartier A Tobelem G Vannier JP Soria J Soria C Cerivastatin, an inhibitor of HMG-CoA reductase, inhibits urokinase/urokinase-receptor expression and MMP-9 secretion by peripheral blood monocytes - A possible protective mechanism against atherothrombosis Thromb Haemostasis 2000 84 680 688 11057870

[B17] Istvan ES Palnitkar M Buchanan SK Deisenhofer J Crystal structure of the catalytic portion of human HMG-CoA reductase: insights into regulation of activity and catalysis Embo Journal 2000 19 819 830 10.1093/emboj/19.5.819 10698924 PMC305622

[B18] Guo J Zhan S Somers J Westenbroek RE Catterall WA Roach DE Sheldon RS Lees-Miller JP Li P Shimoni Y Duff HJ Decrease in density of I-Na is in the common final pathway to heart block in murine hearts overexpressing calcineurin Am J Physiol-Heart C 2006 291 H2669 H2679 10.1152/ajpheart.01247.2005 16751287

[B19] Mocanu MM Gadgil S Yellon DM Baxter GF Mibefradil, a T-type and L-type calcium channel blocker, limits infarct size through a glibenclamide-sensitive mechanism Cardiovasc Drug Ther 1999 13 115 122 10.1023/A:1007732025184 10372226

[B20] Monnier N Procaccio V Stieglitz P Lunardi J Malignant-hyperthermia susceptibility is associated with a mutation of the alpha(1)-subunit of the human dihydropyridine-sensitive L-type voltage-dependent calcium-channel receptor in skeletal muscle Am J Hum Genet 1997 60 1316 1325 10.1086/515454 9199552 PMC1716149

[B21] Testa L Bhindi R Agostoni P Abbate A Zoccai GGLB van Gaal WJ The direct thrombin inhibitor ximelagatran/melagatran: a systematic review on clinical applications and an evidence based assessment of risk benefit profile Expert Opin Drug Saf 2007 6 397 406 10.1517/14740338.6.4.397 17688383

[B22] Iyengar R Boran ADW Systems approaches to polypharmacology and drug discovery Curr Opin Drug Disc 2010 13 297 309 PMC3068535 20443163

[B23] Feuerstein GZ Zaleska MM Krams M Wang X Day M Rutkowski JL Finklestein SP Pangalos MN Poole M Stiles GL Missing steps in the STAIR case: a Translational Medicine perspective on the development of NXY-059 for treatment of acute ischemic stroke J Cerebr Blood F Met 2008 28 217 219 10.1038/sj.jcbfm.9600516 17579658

[B24] Le L Lee EH Hardy DJ Truong TN Schulten K Molecular Dynamics Simulations Suggest that Electrostatic Funnel Directs Binding of Tamiflu to Influenza N1 Neuraminidases Plos Comput Biol 2010 6 9 pii:e1000939 10.1371/journal.pcbi.1000939 PMC2944783 20885781

[B25] Sun H Jiang YJ Yu QS Luo CC Zou JW Effect of mutation K85R on GSK-3 beta: Molecular dynamics simulation Biochem Bioph Res Co 2008 377 962 965 10.1016/j.bbrc.2008.10.096 18955029

[B26] Heinz S Haehnel V Karaghiosoff M Schwarzfischer L Muller M Krause SW Rehli M Species-specific regulation of Toll-like receptor 3 genes in men and mice J Biol Chem 2003 278 21502 21509 10.1074/jbc.M301476200 12672806

[B27] Fraenkel E Odom DT Dowell RD Jacobsen ES Gordon W Danford TW MacIsaac KD Rolfe PA Conboy CM Gifford DK Tissue-specific transcriptional regulation has diverged significantly between human and mouse Nat Genet 2007 39 730 732 10.1038/ng2047 17529977 PMC3797512

[B28] Musshoff F Illegal or legitimate use? Precursor compounds to amphetamine and methamphetamine Drug Metab Rev 2000 32 15 44 10.1081/DMR-100100562 10711406

[B29] Cody JT Precursor medications as a source of methamphetamine and/or amphetamine positive drug testing results J Occup Environ Med 2002 44 435 450 10.1097/00043764-200205000-00012 12024689

[B30] Kraemer T Roditis SK Peters FT Maurer HH Amphetamine concentrations in human urine following single-dose administration of the calcium antagonist prenylamine - Studies using fluorescence polarization immunoassay (FPIA) and GC-MS J Anal Toxicol 2003 27 68 73 12669999 10.1093/jat/27.2.68

[B31] Wishart DS Knox C Guo AC Cheng D Shrivastava S Tzur D Gautam B Hassanali M DrugBank: a knowledgebase for drugs, drug actions and drug targets Nucleic Acids Res 2008 36 D901 D906 18048412 10.1093/nar/gkm958 PMC2238889

[B32] Salwinski L Miller CS Smith AJ Pettit FK Bowie JU Eisenberg D The Database of Interacting Proteins: 2004 update Nucleic Acids Res 2004 32 D449 D451 10.1093/nar/gkh086 14681454 PMC308820

[B33] Alfarano C Andrade CE Anthony K Bahroos N Bajec M Bantoft K Betel D Bobechko B Boutilier K Burgess E The Biomolecular Interaction Network Database and related tools 2005 update Nucleic Acids Res 2005 33 D418 D424 15608229 10.1093/nar/gki051 PMC540005

[B34] Pandey A Mishra GR Suresh M Kumaran K Kannabiran N Suresh S Bala P Shivakumar K Anuradha N Reddy R Human protein reference database - 2006 update Nucleic Acids Res 2006 34 D411 D414 10.1093/nar/gkj141 16381900 PMC1347503

[B35] Tyers M Stark C Breitkreutz BJ Reguly T Boucher L Breitkreutz A BioGRID: a general repository for interaction datasets Nucleic Acids Res 2006 34 D535 D539 10.1093/nar/gkj109 16381927 PMC1347471

[B36] Zanzoni A Montecchi-Palazzi L Quondam M Ausiello G Helmer-Citterich M Cesareni G MINT: a Molecular INTeraction database Febs Lett 2002 513 135 140 10.1016/S0014-5793(01)03293-8 11911893

[B37] Kerrien S Alam-Faruque Y Aranda B Bancarz I Bridge A Derow C Dimmer E Feuermann M Friedrichsen A Huntley R IntAct - open source resource for molecular interaction data Nucleic Acids Res 2007 35 D561 D565 10.1093/nar/gkl958 17145710 PMC1751531

[B38] Shannon P Markiel A Ozier O Baliga NS Wang JT Ramage D Amin N Schwikowski B Ideker T Cytoscape: A software environment for integrated models of biomolecular interaction networks Genome Res 2003 13 2498 2504 10.1101/gr.1239303 14597658 PMC403769

[B39] Muchir A Pavlidis P Decostre V Herron AJ Arimura T Bonne G Worman HJ Activation of MAPK pathways links LMNA mutations to cardiomyopathy in Emery-Dreifuss muscular dystrophy Journal of Clinical Investigation 2007 117 1282 1293 10.1172/JCI29042 17446932 PMC1849984

[B40] Blekhman R Oshlack A Chabot AE Smyth GK, Gilad Y Gene Regulation in Primates Evolves under Tissue-Specific Selection Pressures Plos Genet 2008 4 11 e1000271 10.1371/journal.pgen.1000271 19023414 PMC2581600

[B41] Zhao Y Sheng Z Huang J A systematic analysis of heart transcriptome highlights divergent cardiovascular disease pathways between animal models and humans Mol Biosyst 2012 8 504 510 10.1039/c1mb05415e 22159153

[B42] Berman HM Westbrook J Feng Z Gilliland G Bhat TN Weissig H Shindyalov IN Bourne PE The Protein Data Bank Nucleic Acids Res 2000 28 235 242 10.1093/nar/28.1.235 10592235 PMC102472

[B43] Sali A Blundell TL Comparative Protein Modeling by Satisfaction of Spatial Restraints J Mol Biol 1993 234 779 815 10.1006/jmbi.1993.1626 8254673

[B44] Mackerell AD Feig M Brooks CL Extending the treatment of backbone energetics in protein force fields: Limitations of gas-phase quantum mechanics in reproducing protein conformational distributions in molecular dynamics simulations J Comput Chem 2004 25 1400 1415 10.1002/jcc.20065 15185334

[B45] Buck M Bouguet-Bonnet S Pastor RW MacKerell AD Importance of the CMAP correction to the CHARMM22 protein force field: Dynamics of hen lysozyme Biophys J 2006 90 L36 L38 10.1529/biophysj.105.078154 16361340 PMC1367299

[B46] Jorgensen WL Chandrasekhar J Madura JD Impey RW Klein ML Comparison of Simple Potential Functions for Simulating Liquid Water J Chem Phys 1983 79 926 935 10.1063/1.445869

[B47] Wu GS Robertson DH Brooks CL Vieth M Detailed analysis of grid-based molecular docking: A case study of CDOCKER - A CHARMm-based MD docking algorithm J Comput Chem 2003 24 1549 1562 10.1002/jcc.10306 12925999

[B48] Koska J Spassov VZ Maynard AJ Yan L Austin N Flook PK Venkatachalam CM Fully Automated Molecular Mechanics Based Induced Fit Protein-Ligand Docking Method J Chem Inf Model 2008 48 1965 1973 10.1021/ci800081s 18816046

[B49] Huang JF Li GH CMASA: an accurate algorithm for detecting local protein structural similarity and its application to enzyme catalytic site annotation Bmc Bioinformatics 2010 11 439 10.1186/1471-2105-11-439 PMC2936402 20796320

[B50] Andreeva A Howorth D Brenner SE Hubbard TJP Chothia C Murzin AG SCOP database in 2004: refinements integrate structure and sequence family data Nucleic Acids Res 2004 32 D226 D229 10.1093/nar/gkh039 14681400 PMC308773

[B51] Jorgensen WL Tirado-Rives J Contribution of conformer focusing to the uncertainty in predicting free energies for protein-ligand binding J Med Chem 2006 49 5880 5884 10.1021/jm060763i 17004703

