# Supplementary material for: A comparative analysis of protein targets of withdrawn cardiovascular drugs in human and mouse
Source: J Clin Bioinforma. 2012 May 1;2:10. doi: 10.1186/2043-9113-2-10 (PMC3413526; doi:10.1186/2043-9113-2-10)
Supplement: Additional file 1 — Supporting Information. [file 2043-9113-2-10-S1.doc]

**Supporting Information for**

A comparative analysis of protein targets of withdrawn cardiovascular drugs in human and mouse

Yuqi Zhao^, a^, c Yanjie Wang^, a^, b Jingfei Huang ^a, d, *^

^a^ State Key Laboratory of Genetic Resources and Evolution, Kunming Institute of Zoology, Chinese Academy of Sciences, 32, Eastern Jiaochang Road, Kunming, Yunnan 650223, China; ^b^ Key Laboratory of Animal Models and Human Disease Mechanisms of Chinese Academy of Sciences and Yunnan Province, Kunming Institute of Zoology, Chinese Academy of Sciences, Kunming, Yunnan 650223, China; ^c^ Graduate School of Chinese Academy of Sciences, Beijing 100039, China; ^d^ Kunming Institute of Zoology-Chinese University of Hongkong Joint Research Center for Bio-resources and Human Disease Mechanisms, Kunming 650223, China

Contents

SI Results and Discussion 3 - 4

Figure S1 5

Figure S2 5

Figure S3 6 - 7

Table S1 8 - 11

Table S2 12 - 16

Table S3 17 - 22

Table S4 22 - 24

References 24 - 35

**SI Results and Discussion**

**The variation in mode of actions of FDA-Approved drugs is slight.** Atorvastatin (Lipitor, DrugBank ID code: DB01076) is a member of the drug class known as statins and mainly acts as competitive inhibitor of hydroxymethylglutaryl-coenzyme A reductase (PDB ID code: 1DQA), the rate-determining enzyme in cholesterol biosynthesis via the mevalonate pathway. We found that the binding pocket on HMG-CoA reductase was not affected by sequence divergence between mouse and human for there was only one residue substitution of similar amino acid (Fig. S2, A-B). The binding affinity do not change from mouse to human (Table S3 and Materials and Methods; Student's two-sample t-test, p = 0.38). Lipitor also interacts with other two targets, but only Dipeptidyl peptidase 4 (ADABP, PDB ID code: 2QT9) has crystal structure. The computational docking results between ADABP and lipitor show that functionally complementary substitutions (Gly209Ser, Ser406Gly, Ala458Ser and Ser558Val) occur from mouse to human (Fig. S2, C-D). We predicted that these sites coevolved to maintain the catalytic function. As a result, although the spatial arrangement of lipitor has changed, the function as an inhibitor is not affected. As HMG-CoA reductase, the binding affinity of receptor ADABP do not change either (Table S3 and Materials and Methods; Student's two-sample t-test, p =0.77).

Clopidogrel (Plavix, DrugBank ID code: DB00758) is categorized as platelet aggregation inhibitors and acts as antagonist on P2Y purinoceptor 12 (P2Y12, PDB ID code: 1Y9C), which inhibits the adenylyl cyclase second messenger system . The results show that the function of plavix on P2Y12 is not affected for functionally complementary substitutions (Arg166Gln, Lys168Arg, Asp171Asn and Ser271Thr) also occur (Fig. S2, E-F). For the receptor is involved in platelets aggregation and binds to the G proteins, the coevolved microenvironment can adapt to the regulation of ADP or ATP. The binding free energy also indicates that the binding pattern is little affected by sequence divergence (Table S3 and Materials and Methods; Student's two-sample t-test, p =0.06).

**
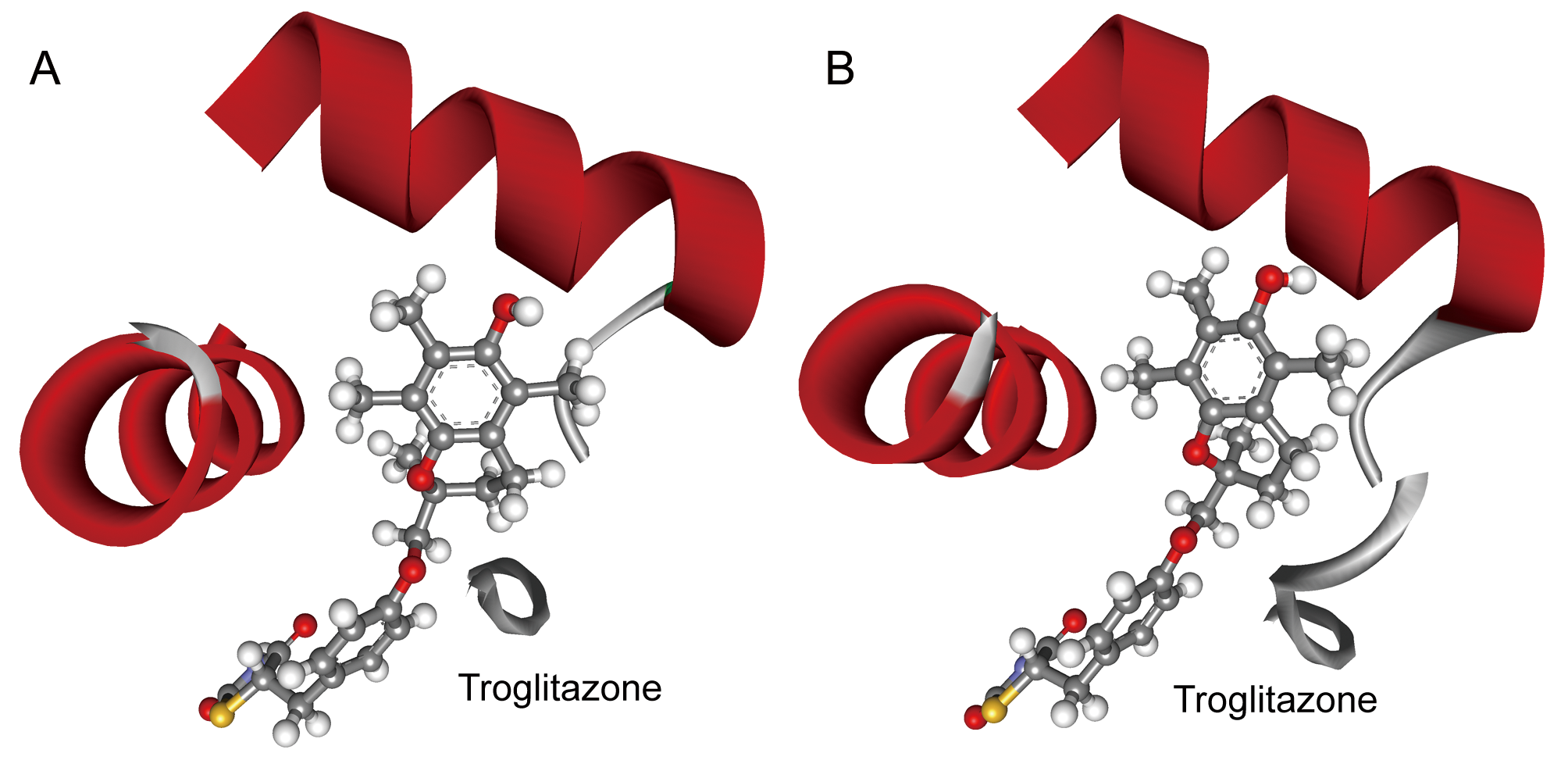
**

***Figure S1.* Binding patterns of troglitazone with steroid hormone receptor ERR1.** Panel (A) illustrates interactions between troglitazone and human Steroid hormone receptor ERR1, while (B) represents interactions between drug and corresponding mouse target. The residue substitutions in the binding pocket are labeled.

**
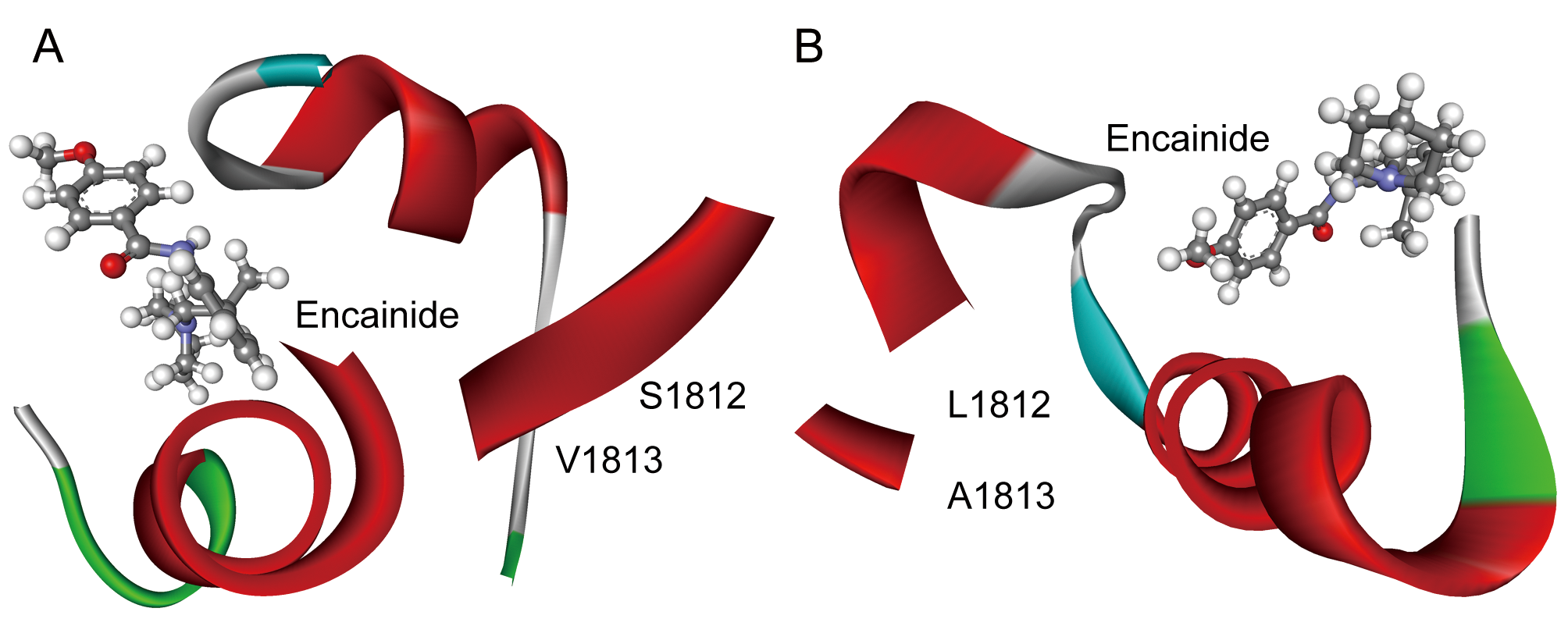
**

***Figure S2.* Binding patterns of encainide with its targets.** Panel (A) illustrates interactions between encainide and human sodium channel protein type 5 subunit alpha, while (B) represents interactions between drug and corresponding mouse target. The residue substitutions in the binding pocket and chains are labeled.

**
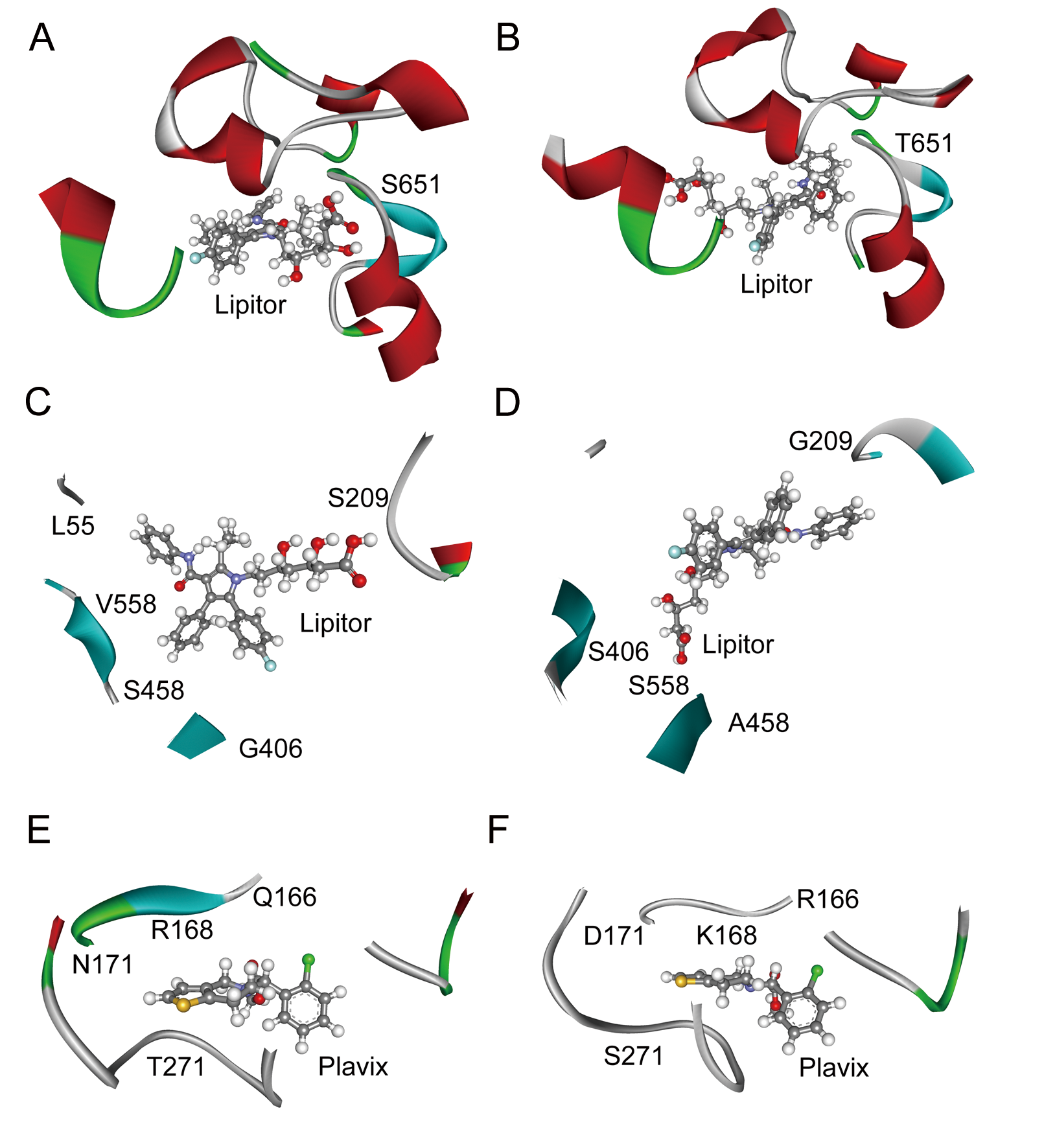
**

***Figure S3.* Binding patterns of FDA-approved drugs with their targets.** Panel (A) illustrates interactions between Atorvastatin and human HMG-CoA reductase, while (B) represents interactions between drug and corresponding mouse target. Panel (C) illustrates interactions between Atorvastatin and human Dipeptidyl peptidase 4, while (D) represents interactions between drug and corresponding mouse target. Panel (E) illustrates interactions between Clopidogrel and human P2Y purinoceptor 12, while (F) represents interactions between drug and corresponding mouse target.The residue substitutions in the binding pocket are labeled.

**Table S1. Table of both withdrawn and two FDA-approved cardiovascular drugs.**

| **DrugBank ID** | Generic Name | Drug Categories | Reasons for Withdrawal | Human Targets | Drug Action | PDB ID | Mouse Orthologs ^b^ | Functional change | References ^c^ |
| --- | --- | --- | --- | --- | --- | --- | --- | --- | --- |
| **DB00197** | Troglitazone | Vasodilator Agents; Platelet Aggregation Inhibitors | hepatotoxicity | P37231 | agonist | 3DZY | P37238 | NO |  |
|  |  |  |  | O60488 | antagonist | Null | Q5D071 | NO |  |
|  |  |  |  | P05121 | antagonist | 1C5G | P22777 | NO |  |
|  |  |  |  | Q99808 | antagonist | Null | Q9JIM1 | NO |  |
|  |  |  |  | P62508 | inverse agonist | 2ZKC | P62509 | NO |  |
|  |  |  |  | P11474 | inverse agonist | 3D24 | O08580 | NO |  |
| **DB00439** | Cerivastatin | Anticholesteremic Agents; Antilipemic Agents | fatal Rhabdomyolysis | P04035 | antagonist | 1HWL | Q01237 | NO |  |
| **DB01228** | Encainide | Antiarrhythmic Agents | frequent proarrhythmic | Q14524 | blocker | 2KBI | Q9JJV9 | NO |  |
| **DB01388** | Mibefradil | Antihypertensive Agents; Calcium Channel Blockers | harmful interactions with other drugs | O43497 | antagonist | Null | Q5SUF7 | NO |  |
|  |  |  |  | O95180 | antagonist | Null | O88427 | NO |  |
|  |  |  |  | Q13936 | antagonist | 3G43 | Q5S8D1 | NO |  |
|  |  |  |  | Q01668 | antagonist | 3LV3 | Q99246 | NO |  |
|  |  |  |  | O60840 | antagonist | Null | Q7TNI3 | NO |  |
|  |  |  |  | Q9P0X4 | antagonist | Null | E0CXK2 | NO |  |
|  |  |  |  | Q13698 | antagonist | 2VAY | Q02789 | NO |  |
|  |  |  |  | Q02641 | antagonist | Null | Q8R3Z5 | NO |  |
|  |  |  |  | Q08289 | antagonist | Null | Q8CC27 | NO |  |
|  |  |  |  | P54284 | antagonist | Null | P54285 | NO |  |
|  |  |  |  | O00305 | antagonist | 2D46 | Q8R0S4 | NO |  |
| **DB04825** | Prenylamine | Vasodilator Agents; Calcium Channel Blockers | cardiac arrhythmias | P62158 | blocker | 2F3Y | P62204 | NO | null |
| **DB04831** | Ticrynafen | Antihypertensive Agents; Diuretics | hepatitis | null | null | Null | null | null | null |
| **DB04898** | Ximelagatran | Anticoagulants; Antithrombotic Agents | hepatotoxicity | P00734 | Antagonist; inhibitor | 2BVR | P19221 | NO |  |
| **DB01076^a^** | Atorvastatin | Anticholesteremic Agents; HMG-CoA Reductase Inhibitors; Hydroxymethylglutaryl-CoA Reductase Inhibitors | null | P04035 | antagonist | 1DQA | Q01237 | NO |  |
|  |  |  |  | P27487 | inhibitor | 2QT9 | P28843 | NO |  |
|  |  |  |  | P35869 | agonist | null | P30561 | NO |  |
| **DB00758^a^** | Clopidogrel | Antiplatelet Agents; Fibrinolytic Agents; Platelet Aggregation Inhibitors | null | Q9H244 | antagonist | 1Y9C | Q9CPV9 | NO |  |
|  | **Note: ^a^ The red DrugBank IDs represent the FDA-approved drugs; ^b^ The corresponding mouse drug targets were identified through reciprocal BLAST; ^c^ References for** **pharmacological studies in mice.** | | | | | | | | |

Table S2. Interactions of withdrawn cardiovascular drug targets.

| **Uniprot_Acs^a^** | **Gene_Symbls** | | | **Interactions^b^** | **Main_References^c^** | |
| --- | --- | --- | --- | --- | --- | --- |
| [P37231](http://www.uniprot.org/uniprot/P37231) | | PPARG | EDF1,NCOA1,MED1,SYT1,HDAC3,RXRB,SMRD3,PRGC1,NCOR1,HMGA1,TDIF2,GA45G,MK08,NRIP1,FABPL,CNOT1,RXRA,NCOR2,NCOA3,CSN5,PR285,NCOA6,NF2L2,FOXO1,NR0B1,NCOA2,KIF1A,NCOA4,RXRG,RANB9,NR0B2,MK01,MED24,BRD8,GA45B | |  |  |
| [O60488](http://www.uniprot.org/uniprot/O60488) | | **ACSL4** | No experimental data | | Null |  |
| [P05121](http://www.uniprot.org/uniprot/P05121) | | **SERPINE1** | LRP1,F2,ACTN4,ORM1,KLK2,PLAU,VTN,KRT18,IGFBP5,LRP1B,ELANE,PLG,LRP2,MMP3,PLAT,UBQLN4 | |  |  |
| [Q99808](http://www.uniprot.org/uniprot/Q99808) | | **SLC29A1** | STK19 | |  |  |
| [P62508](http://www.uniprot.org/uniprot/P62508) | | **ESRRG** | NR0B2,NCOA1,PNRC2,ERR1,PRGC1,PROX1,DAXX,PNRC1,CALM,ARHGF,DUT,PRGC2,NRIP1 | |  |  |
| [P11474](http://www.uniprot.org/uniprot/P11474) | | **ESRRA** | EXOS6,LYST,NCOA3,PRGC1,NR0B1,KPCD,NR0B2,PNRC2,PROX1,DUT,ESR1,TF2B,ERR3,NCOA1 | |  |  |
| [P04035](http://www.uniprot.org/uniprot/P04035) | | **HMGCR** | KAPCA,RHG01,STA13,INSI1 | |  |  |
| [Q14524](http://www.uniprot.org/uniprot/Q14524) | | **SCN5A** | SNTB2,RGS3,NED4L,CBL,SNTG2,DLG1,INADL,DLG4,RGS2,DLG2,SNTB1,SNTA1,DLG3 | |  |  |
| [O43497](http://www.uniprot.org/uniprot/O43497) | | **CACNA1G** | UBQLN4 | |  |  |
| [O95180](http://www.uniprot.org/uniprot/O95180) | | **CACNA1H** | GNG2,KCNMA1 | |  |  |
| [Q13936](http://www.uniprot.org/uniprot/Q13936) | | **CACNA1C** | PPM1A,RYR2,GNB1,CABP1,SRI,PRKACA,CACNB3,RIMS1 | |  |  |
| [Q01668](http://www.uniprot.org/uniprot/Q01668) | | **CACNA1D** | FGFR2,STX1A | |  |  |
| [O60840](http://www.uniprot.org/uniprot/O60840) | | **CACNA1F** | CABP4 | |  |  |
| [Q9P0X4](http://www.uniprot.org/uniprot/Q9P0X4) | | **CACNA1I** | No experimental data | | Null |  |
| [Q13698](http://www.uniprot.org/uniprot/Q13698) | | **CACNA1S** | RYR1,SRI,RIMS2  S12 | |  |  |
| [Q02641](http://www.uniprot.org/uniprot/Q02641) | | **CACNB1** | ATN1,DYNLL1,CACNA1A,REM1 | |  |  |
| [Q08289](http://www.uniprot.org/uniprot/Q08289) | | **CACNB2** | PRKACA,REM1 | |  |  |
| [P54284](http://www.uniprot.org/uniprot/P54284) | | **CACNB3** | CACNA1B,CACNA1C,SYT1 | |  |  |
| [O00305](http://www.uniprot.org/uniprot/O00305) | | **CACNB4** | TBL3,MED31,MAP1A,CACNA1A,SYT1,REM1,PTN | |  |  |
| [P62158](http://www.uniprot.org/uniprot/P62158) | | **CALM1** | GLP2R,CP110,LYST,MYF6,FAS,OPRM1,KCNQ5,MYOD1,GRK4,MARCKS,SCTR,GRIN1,RGS4,KCNQ3,CAMK1,TCF3,PLCB3,PHKG1,RGS10,RELA,AKAP5,VIPR1,MYF5,KIAA1683,RRAD,AKAP9,STRN,NR3C1,GRB7,MYO7A,ITPKA,FER,PTH2R,MYO6,ADD2,MAPT,CLTB,STRN4,CALD1,CSNK2A1P,TNNI2,KCNN2,MYLK,GRM5,GRM7,HSP90AA1,PCNT,SNTA1,ADD1,PLCD1,MINK1,KRAS,ESRRG,CRHR1,TRPC3,NRGN,ATP2B1,IQCB1,PTPRA,RAB3B,GRM4,SNCA,TRPV1,CABIN1,RALA,GRK1,MYO9B,MYOG,RIT2,ADCY8,CAMK2G,CALCR,EGFR,IQGAP2,ADCYAP1R1,KCNN4,CNGA2,PPEF1,MIP,NEUROD1,IQGAP1,EWSR1,PPEF2,SYT1,GLP1R,TRPV4,LTF,MRPL20,KCNQ2,CSNK2A1,YWHAE,PCP4,HMMR,RGS2,DLG1,CSNK2A2,DLG3,HTR2C,RYR1,CAMKK2,ASCL2,MYO10,GRM3,OBSCN,PDE1A,HRAS,CAMKK1,ESR1,RALB,REL,TTN,AR,GAP43,TCF4,TRPV6,CNN1,STRN3,EDF1,PIK3C3,DDX5,INSR,CCND1,ITPKB,ESR2,CCNE1,GJB1  S13 | |  |  |
| [P00734](http://www.uniprot.org/uniprot/P00734) | | **F2** | GGCX,SERPING1,THBS1,F9,KNG1,FGA,F5,PROC,F2RL2,CPB2,F2RL1,SERPIND1,F8,PROZ,SERPINA5,GP5,ITGA2B,C6,HGFAC,THBD,F2RL3,IGFBP3,F13A1,GP1BA,SERPINE2,F2R,SERPINE1,SERPINB6,SERPINC1,PLAU,AMBP,PROS1,ST13,AANAT,IGFBP5,AKR7A2,F11,SPP1 | |  |  |

Note: ^a^ Uniprot Accessions of withdrawn cardiovascular drug targets in human; ^b^ Interactions of drug targets that were proved by experiments; ^c^ main references for the interactions.

**Table S3. The binding free energies of withdrawn cardiovascular drugs to their targets.**

| **DrugBank_ID** | Target_Uniprot_Accession | ΔGh(kcal/mol) | Mouse_Uniprot_ACs | ΔGm(kcal/mol) |
| --- | --- | --- | --- | --- |
| **DB00197** | P37231 | -38.69054 | P37238 | -52.68061 |
|  |  | -32.76336 |  | -54.76857 |
|  |  | -38.43798 |  | -70.7862 |
|  |  | -29.54503 |  | -63.05442 |
|  |  | -61.48121 |  | -61.47478 |
|  |  | -52.20001 |  | -75.92494 |
|  |  | -47.23538 |  | -82.25261 |
|  |  | -29.51982 |  | -51.66813 |
|  |  | -32.01089 |  | -76.82899 |
|  |  | -15.07131 |  | -60.70904 |
| **DB00197** | P05121 | -25.42788 | P22777 | 360.47033 |
|  |  | -7.10415 |  | 362.52442 |
|  |  | -21.59473 |  | null |
|  |  | -45.82581 |  | null |
|  |  | -67.81256 |  | null |
|  |  | -31.15524 |  | null |
|  |  | -16.60648 |  | null |
|  |  | -13.59952 |  | null |
|  |  | -32.17905 |  | null |
|  |  | -1.0656 |  | null |
| **DB00197** | P11474 | -1.55332 | O08580 | -17.15659 |
|  |  | -36.87147 |  | -15.23053 |
|  |  | -40.6824 |  | -32.43063 |
|  |  | -40.88475 |  | -23.9473 |
|  |  | -31.65251 |  | -38.15807 |
|  |  | -40.80003 |  | -26.92835 |
|  |  | -18.14741 |  | -28.8662 |
|  |  | -43.59559 |  | -11.46258 |
|  |  | -30.09641 |  | -23.93459 |
|  |  | -34.27226 |  | 6.0553 |
| **DB00439** | P04035 | -60.7405 | Q01237 | -84.29649 |
|  |  | -36.47969 |  | -95.81546 |
|  |  | -12.34967 |  | -64.76912 |
|  |  | -26.48913 |  | -46.63295 |
|  |  | -64.76912 |  | -73.28244 |
|  |  | -46.63295 |  | -62.62469 |
|  |  | -23.28244 |  | -34.7419 |
|  |  | -62.62469 |  | -10.41355 |
|  |  | -1.41355 |  | -63.2292 |
|  |  | -34.7419 |  | -76.19135 |
| **DB01228** | Q14524 | -10.26342 | Q9JJV9 | -22.73252 |
|  |  | -6.88953 |  | -43.38533 |
|  |  | -7.25358 |  | -62.00937 |
|  |  | -12.25115 |  | -34.12797 |
|  |  | -26.65292 |  | -32.60488 |
|  |  | -23.58283 |  | -12.36797 |
|  |  | -59.49255 |  | -27.46929 |
|  |  | -35.3862 |  | -29.68971 |
|  |  | -26.89964 |  | -15.02219 |
|  |  | -44.35878 |  | -9.08234 |
| **DB01388** | Q13936 | -28.66038 | Q5S8D1 | -118.27119 |
|  |  | -65.51403 |  | -91.64422 |
|  |  | -81.0144 |  | -97.11656 |
|  |  | -57.15907 |  | -151.08308 |
|  |  | -77.28528 |  | -136.59989 |
|  |  | -46.09379 |  | -99.1693 |
|  |  | -49.89369 |  | -146.93643 |
|  |  | -47.78067 |  | -82.80422 |
|  |  | -0.98414 |  | -82.8036 |
|  |  | -48.52884 |  | -82.80326 |
| **DB04898** | P00734 | -21.48221 | P19221 | -40.09378 |
|  |  | -49.42784 |  | -78.39641 |
|  |  | -22.13548 |  | -41.60142 |
|  |  | -45.55339 |  | -70.75706 |
|  |  | -39.99807 |  | -33.49798 |
|  |  | -46.15591 |  | -45.99742 |
|  |  | -45.36475 |  | -92.4379 |
|  |  | -28.56567 |  | -56.6732 |
|  |  | -13.61795 |  | -77.69347 |
|  |  | -33.59445 |  | -53.7996 |
| **DB01076** | P04035 | -57.57159 | Q01237 | -6.03258 |
|  |  | -33.49904 |  | 3.30783 |
|  |  | -12.79071 |  | -42.72987 |
|  |  | -46.59826 |  | -54.05494 |
|  |  | -45.73702 |  | -76.72214 |
|  |  | -48.86167 |  | -12.10339 |
|  |  | -61.76703 |  | -46.53311 |
|  |  | -44.33487 |  | -36.96976 |
|  |  | -52.931 |  | -37.85539 |
|  |  | -39.93514 |  | -54.37622 |
| **DB01076** | P27487 | -46.52245 | P28843 | -61.07224 |
|  |  | -37.60097 |  | -85.5591 |
|  |  | -13.16038 |  | -6.18418 |
|  |  | -60.74861 |  | -76.99902 |
|  |  | -64.75766 |  | -39.85214 |
|  |  | -45.28082 |  | -7.66664 |
|  |  | -50.72475 |  | -73.94847 |
|  |  | -53.31079 |  | -83.13868 |
|  |  | -87.11792 |  | -43.07056 |
|  |  | -72.31572 |  | -19.85196 |
| **DB00758** | Q9H244 | -44.30476 | Q9CPV9 | -59.06068 |
|  |  | -39.42008 |  | -72.43849 |
|  |  | -41.46136 |  | -45.88875 |
|  |  | -36.26717 |  | -33.55748 |
|  |  | -33.48318 |  | -33.23858 |
|  |  | -35.82651 |  | -31.75632 |
|  |  | -35.88891 |  | -68.11231 |
|  |  | -33.99152 |  | -45.34534 |
|  |  | -39.1443 |  | -63.25149 |
|  |  | -28.32048 |  | -42.83864 |
|  |  |  |  |  |

Note: ΔGm denotes binding energies of mouse drug-receptor while ΔGh denotes binding energies of human drug-receptor.

**Table S4. Binding sites of withdrawn cardiovascular drugs and potential off-targets.**

| **Human Targets** | **Binding Sites in Human Target** | **Human Potential Off-Targets** | **Human Potential Off-Target Structures** | **Mouse Drug Targets** | **Drug Binding Sites in Mouse Target** | **Mouse Potential Off-Targets** |
| --- | --- | --- | --- | --- | --- | --- |
| **P37231** | CYS285; SER289; ILE326; LEU330; LEU469; TYR473 | NAD(P)-binding Rossmann-fold domains; Multiheme cytochromes; PLP-dependent transferases; Metallo-dependent hydrolases; P-loop containing nucleoside triphosphate hydrolases; Zn-dependent exopeptidases; Ferritin-like | 1oc2; 1fs7; 2byl; 2imr; 1lkx; 2c1c;  2oc5 | P37238 | LYS301; ILE307; LEU309; ASP310; LEU311; GLN314; VAL315 | null |
| **P11474** | PRO206; LEU209; GLU235; VAL238; VAL268; ARG276; TYR326 | null | null | O08580 | PRO206; GLU207; LEU209; VAL268; LEU269; ARG276; TYR326; LYS330 | null |
| **P05121** | HIS208; ARG209; ARG210; LEU211; PHE212; LYS214; GLU401 | null | null | P22777 | GLN34; TYR60; SER63; SER64; THR94; THR116; PHE140 | null |
| **Q14524** | SER1834; ASN1837; MET1838; ASP1839; LYS1859; ARG1860; SER1865 | null | null | Q9JJV9 | MET1838; ASP1839; PRO1841; ASP1852; PHE1855; ALA1856; LYS1859 | null |
| **P04035** | ALA564; ASN567; ARG568; ARG571; VAL720; LYS722 | YnzC-like | 2hep | Q01237 | CYS561; ALA564; SER565; ASN567; ARG568; ARG571; VAL720; SER852; ASN529 | null |
| **Q13936** | ARG1643; THR1644; LYS1647; ARG1615; LEU1618; ARG1619; GLU1623 | null | null | Q5S8D1 | ILE1640; ARG1643; THR1644; MET1646; ARG1615; LEU1618; ARG1619; GLU1623 | null |
| **P00734** | ILE174; ASP189; GLU192; GLU217; ARG221; GLY226; PHE227 | null | null | P19221 | HIS57; ASN98; ILE174; ASP189; SER195; TRP215; GLY219 | null |

**References**

1. Gladding PA, Webster MWI, Zeng ISL, Stewart J, Ruygrok P, Ormiston J, Farrell H, Ei-Jack S, Armstrong G, Kay P, et al: **The pharmacogenetics and pharmacodynamics of clopidogrel response: An analysis from the PRINC (Plavix response in coronary intervention) trial.** *J Am Coll Cardiol* 2008, **51:**A333-A333.

2. Young PW, Buckle DR, Cantello BCC, Chapman H, Clapham JC, Coyle PJ, Haigh D, Hindley RM, Holder JC, Kallender H, et al: **Identification of high-affinity binding sites for the insulin sensitizer rosiglitazone (BRL-49653) in rodent and human adipocytes using a radioiodinated ligand for peroxisomal proliferator-activated receptor gamma.** *J Pharmacol Exp Ther* 1998, **284:**751-759.

3. Staruschenko A, Pavlov TS, Levchenko V, Karpushev AV, Vandewalle A: **Peroxisome Proliferator-Activated Receptor gamma Antagonists Decrease Na(+) Transport via the Epithelial Na(+) Channel.** *Mol Pharmacol* 2009, **76:**1333-1340.

4. Shibata T, Matsui K, Nagao K, Shinkai H, Yonemori F, Wakitani K: **Pharmacological profiles of a novel oral antidiabetic agent, JTT-501, an isoxazolidinedione derivative.** *European Journal of Pharmacology* 1999, **364:**211-219.

5. Shimizu K, Aikawa M, Takayama K, Libby P, Mitchell RN: **Direct anti-inflammatory mechanisms contribute to attenuation of experimental allograft arteriosclerosis by statins.** *Circulation* 2003, **108:**2113-2120.

6. Lawler OA, Miggin SM, Kinsella BT: **The effects of the statins lovastatin and cerivastatin on signalling by the prostanoid IP-receptor.** *Brit J Pharmacol* 2001, **132:**1639-1649.

7. Gomoll AW, Byrne JE, Mayol RF: **Comparative Antiarrhythmic Actions of Encainide and Its Major Metabolites.** *Arch Int Pharmacod T* 1986, **281:**277-297.

8. Roden DM, Wood AJJ, Wilkinson GR, Woosley RL: **Disposition Kinetics of Encainide and Metabolites.** *Am J Cardiol* 1986, **58:**C4-C9.

9. Yang T, Snyders D, Roden DM: **Drug block of I-kappa r: Model systems and relevance to human arrhythmias.** *J Cardiovasc Pharm* 2001, **38:**737-744.

10. Himmel HM, Stengel W, Ravens U: **Selectivity of blocking of low- versus high-voltage activated calcium currents by the dihydropyridine derivatives Bay E5759 and Bay A4339 in neuroblastoma-glioma NG 108-15 cells.** *Pharmacol Res* 2001, **44:**113-116.

11. Larsson M, Ahnoff M, Abrahamsson A, Logren U, Fakt C, Ohrman I, Persson BA: **Determination of ximelagatran, an oral direct thrombin inhibitor, its active metabolite melagatran, biological samples by liquid and the intermediate metabolites, in chromatography-mass spectrometry.** *J Chromatogr B* 2003, **783:**335-347.

12. Crowther MA, Weitz JI: **Ximelagatran: the first oral direct thrombin inhibitor.** *Expert Opin Inv Drug* 2004, **13:**403-413.

13. Ishii I, Kitahara M, Kanaki T, Saito Y: **Atherosclerosis induced by chronic inhibition of the synthesis of nitric oxide in moderately hypercholesterolaemic rabbits is suppressed by pitavastatin.** *Brit J Pharmacol* 2010, **159:**1418-1428.

14. Moshfegh K, Redondo M, Julmy F, Wuillemin WA, Gebauer MU, Haeberli A, Meyer BJ: **Antiplatelet effects of clopidogrel compared with aspirin after myocardial infarction: Enhanced inhibitory effects of combination therapy.** *J Am Coll Cardiol* 2000, **36:**699-705.

15. Evangelista V, Manarini S, Dell'Elba G, Martelli N, Napoleone E, Di Santo A, Savi P, Lorenzet R: **Clopidogrel inhibits platelet-leukocyte adhesion and platelet-dependent leukocyte activation.** *Thromb Haemostasis* 2005, **94:**568-577.

16. Zhang K, Wang S, Malhotra J, Hassler JR, Back SH, Wang G, Chang L, Xu W, Miao H, Leonardi R, et al: **The unfolded protein response transducer IRE1alpha prevents ER stress-induced hepatic steatosis.** *EMBO J* 2011.

17. Yim MJ, Hosokawa M, Mizushina Y, Yoshida H, Saito Y, Miyashita K: **Suppressive Effects of Amarouciaxanthin A on 3T3-L1 Adipocyte Differentiation through Down-regulation of PPARgamma and C/EBPalpha mRNA Expression.** *J Agric Food Chem* 2011, **59:**1646-1652.

18. Yeo J, Kang YM, Cho SI, Jung MH: **Effects of a multi-herbal extract on type 2 diabetes.** *Chin Med* 2011, **6:**10.

19. Werner C, Gensch C, Poss J, Haendeler J, Bohm M, Laufs U: **Pioglitazone activates aortic telomerase and prevents stress-induced endothelial apoptosis.** *Atherosclerosis* 2011.

20. Thiagarajan RD, Georgas KM, Rumballe BA, Lesieur E, Chiu HS, Taylor D, Tang DT, Grimmond SM, Little MH: **Identification of Anchor Genes during Kidney Development Defines Ontological Relationships, Molecular Subcompartments and Regulatory Pathways.** *Plos One* 2011, **6:**e17286.

21. Swarnkar G, Sharan K, Siddiqui JA, Chakravarti B, Rawat P, Kumar M, Arya KR, Maurya R, Chattopadhyay N: **A novel flavonoid isolated from the steam-bark of Ulmus Wallichiana Planchon stimulates osteoblast function and inhibits osteoclast and adipocyte differentiation.** *Eur J Pharmacol* 2011.

22. Sun QC, Xiao JG, Yang XJ, Tian WD: **[Expression profiling of peroxisome proliferation activate receptor gamma2 and phosphorylation during adipogenic differentiation of adipose-derived stem cells].** *Sichuan Da Xue Xue Bao Yi Xue Ban* 2011, **42:**10-14.

23. Sos BC, Harris C, Nordstrom SM, Tran JL, Balazs M, Caplazi P, Febbraio M, Applegate MA, Wagner KU, Weiss EJ: **Abrogation of growth hormone secretion rescues fatty liver in mice with hepatocyte-specific deletion of JAK2.** *J Clin Invest* 2011.

24. Seo JB, Choe SS, Jeong HW, Park SW, Shin HJ, Choi SM, Park JY, Choi EW, Kim JB, Seen DS, et al: **Anti-obesity effects of Lysimachia foenum-graecum characterized by decreased adipogenesis and regulated lipid metabolism.** *Exp Mol Med* 2011.

25. Semirale AA, Zhang X, Wiren KM: **Body composition changes and inhibition of fat development in vivo implicates androgen in regulation of stem cell lineage allocation.** *J Cell Biochem* 2011.

26. Cao H, Anderson RA: **Cinnamon polyphenol extract regulates tristetraprolin and related gene expression in mouse adipocytes.** *J Agric Food Chem* 2011, **59:**2739-2744.

27. Chung MC, Jorgensen SC, Tonry JH, Kashanchi F, Bailey C, Popov S: **Secreted Bacillus anthracis proteases target the host fibrinolytic system.** *FEMS Immunol Med Microbiol* 2011.

28. Croci DO, Cumashi A, Ushakova NA, Preobrazhenskaya ME, Piccoli A, Totani L, Ustyuzhanina NE, Bilan MI, Usov AI, Grachev AA, et al: **Fucans, but Not Fucomannoglucuronans, Determine the Biological Activities of Sulfated Polysaccharides from Laminaria saccharina Brown Seaweed.** *Plos One* 2011, **6:**e17283.

29. Dendooven A, van Oostrom O, van der Giezen DM, Willem Leeuwis J, Snijckers C, Joles JA, Robertson EJ, Verhaar MC, Nguyen TQ, Goldschmeding R: **Loss of endogenous bone morphogenetic protein-6 aggravates renal fibrosis.** *Am J Pathol* 2011, **178:**1069-1079.

30. Filiano AN, Fathallah-Shaykh HM, Fiveash J, Gage J, Cantor A, Kharbanda S, Johnson MR: **Gene Expression Analysis in Radiotherapy Patients and C57BL/6 Mice as a Measure of Exposure to Ionizing Radiation.** *Radiat Res* 2011.

31. Hua H, Zhang R, Yu S, Wang H, Zhao Y, Li W: **Tissue-type plasminogen activator depletion affects the nasal mucosa matrix reconstruction in allergic rhinitis mice.** *Allergol Immunopathol (Madr)* 2011.

32. Lopez-Guisa JM, Rassa AC, Cai X, Collins SJ, Eddy AA: **Vitronectin accumulates in the interstitium but minimally impacts fibrogenesis in experimental chronic kidney disease.** *Am J Physiol Renal Physiol* 2011.

33. Matsuzaki S, Botchorishvili R, Jardon K, Maleysson E, Canis M, Mage G: **Impact of intraperitoneal pressure and duration of surgery on levels of tissue plasminogen activator and plasminogen activator inhibitor-1 mRNA in peritoneal tissues during laparoscopic surgery.** *Hum Reprod* 2011.

34. Mutoh M, Teraoka N, Takasu S, Takahashi M, Onuma K, Yamamoto M, Kubota N, Iseki T, Kadowaki T, Sugimura T, Wakabayashi K: **Loss of adiponectin promotes intestinal carcinogenesis in Min and wild-type mice.** *Gastroenterology* 2011.

35. Sakakibara H, Romanowski CP, Jakubcakova V, Flachskamm C, Shimoi K, Kimura M: **Feeble awake effects of plasminogen activator inhibitor type-1 in mice.** *Behav Brain Res* 2011.

36. SenGupta DJ, Lum PY, Lai Y, Shubochkina E, Bakken AH, Schneider G, Unadkat JD: **A single glycine mutation in the equilibrative nucleoside transporter gene, hENT1, alters nucleoside transport activity and sensitivity to nitrobenzylthioinosine.** *Biochemistry* 2002, **41:**1512-1519.

37. Harewood L, Liu M, Keeling J, Howatson A, Whiteford M, Branney P, Evans M, Fantes J, Fitzpatrick DR: **Bilateral renal agenesis/hypoplasia/dysplasia (BRAHD): postmortem analysis of 45 cases with breakpoint mapping of two de novo translocations.** *Plos One* 2010, **5:**e12375.

38. Arase S, Ishii K, Igarashi K, Aisaki K, Yoshio Y, Matsushima A, Shimohigashi Y, Arima K, Kanno J, Sugimura Y: **Endocrine Disrupter Bisphenol A Increases In Situ Estrogen Production in the Mouse Urogenital Sinus.** *Biol Reprod* 2010.

39. Alaynick WA, Way JM, Wilson SA, Benson WG, Pei L, Downes M, Yu R, Jonker JW, Holt JA, Rajpal DK, et al: **ERRgamma regulates cardiac, gastric, and renal potassium homeostasis.** *Mol Endocrinol* 2010, **24:**299-309.

40. Berry R, Harewood L, Pei L, Fisher M, Brownstein D, Ross A, Alaynick WA, Moss J, Hastie ND, Hohenstein P, et al: **Esrrg functions in early branch generation of the ureteric bud and is essential for normal development of the renal papilla.** *Hum Mol Genet* 2011, **20:**917-926.

41. Rangwala SM, Wang X, Calvo JA, Lindsley L, Zhang Y, Deyneko G, Beaulieu V, Gao J, Turner G, Markovits J: **Estrogen-related receptor gamma is a key regulator of muscle mitochondrial activity and oxidative capacity.** *J Biol Chem* 2010, **285:**22619-22629.

42. Friese A, Kaltschmidt JA, Ladle DR, Sigrist M, Jessell TM, Arber S: **Gamma and alpha motor neurons distinguished by expression of transcription factor Err3.** *Proc Natl Acad Sci U S A* 2009, **106:**13588-13593.

43. Abujarour R, Efe J, Ding S: **Genome-wide gain-of-function screen identifies novel regulators of pluripotency.** *Stem Cells* 2010, **28:**1487-1497.

44. Song G, Wang L: **Nuclear receptor SHP activates miR-206 expression via a cascade dual inhibitory mechanism.** *Plos One* 2009, **4:**e6880.

45. Sanoudou D, Duka A, Drosatos K, Hayes KC, Zannis VI: **Role of Esrrg in the fibrate-mediated regulation of lipid metabolism genes in human ApoA-I transgenic mice.** *Pharmacogenomics J* 2010, **10:**165-179.

46. Hirvonen J, Rajalin AM, Wohlfahrt G, Adlercreutz H, Wahala K, Aarnisalo P: **Transcriptional activity of estrogen-related receptor gamma (ERRgamma) is stimulated by the phytoestrogen equol.** *J Steroid Biochem Mol Biol* 2011, **123:**46-57.

47. Wagatsuma A, Kotake N, Mabuchi K, Yamada S: **Expression of nuclear-encoded genes involved in mitochondrial biogenesis and dynamics in experimentally denervated muscle.** *J Physiol Biochem* 2011.

48. Wagatsuma A, Kotake N, Kawachi T, Shiozuka M, Yamada S, Matsuda R: **Mitochondrial adaptations in skeletal muscle to hindlimb unloading.** *Mol Cell Biochem* 2011, **350:**1-11.

49. Uguccioni G, Hood DA: **The importance of PGC-1alpha in contractile activity-induced mitochondrial adaptations.** *Am J Physiol Endocrinol Metab* 2011, **300:**E361-371.

50. Ellis JM, Mentock SM, Depetrillo MA, Koves TR, Sen S, Watkins SM, Muoio DM, Cline GW, Taegtmeyer H, Shulman GI, et al: **Mouse cardiac acyl coenzyme a synthetase 1 deficiency impairs Fatty Acid oxidation and induces cardiac hypertrophy.** *Mol Cell Biol* 2011, **31:**1252-1262.

51. Workman A, Jones C: **Productive infection and bICP0 early promoter activity of bovine herpesvirus 1 are stimulated by E2F1.** *J Virol* 2010, **84:**6308-6317.

52. Wilson BJ, Tremblay AM, Deblois G, Sylvain-Drolet G, Giguere V: **An acetylation switch modulates the transcriptional activity of estrogen-related receptor alpha.** *Mol Endocrinol* 2010, **24:**1349-1358.

53. Wei W, Wang X, Yang M, Smith LC, Dechow PC, Sonoda J, Evans RM, Wan Y: **PGC1beta mediates PPARgamma activation of osteoclastogenesis and rosiglitazone-induced bone loss.** *Cell Metab* 2010, **11:**503-516.

54. Watson PA, Birdsey N, Huggins GS, Svensson E, Heppe D, Knaub L: **Cardiac-specific overexpression of dominant-negative CREB leads to increased mortality and mitochondrial dysfunction in female mice.** *Am J Physiol Heart Circ Physiol* 2010, **299:**H2056-2068.

55. Wang SC, Myers S, Dooms C, Capon R, Muscat GE: **An ERRbeta/gamma agonist modulates GRalpha expression, and glucocorticoid responsive gene expression in skeletal muscle cells.** *Mol Cell Endocrinol* 2010, **315:**146-152.

56. Sanchez-Gomez MV, Alberdi E, Perez-Navarro E, Alberch J, Matute C: **Bax and calpain mediate excitotoxic oligodendrocyte death induced by activation of both AMPA and kainate receptors.** *J Neurosci* 2011, **31:**2996-3006.

57. Huang B, Yuan HD, Kim DY, Quan HY, Chung SH: **Cinnamaldehyde Prevents Adipocyte Differentiation and Adipogenesis via Regulating PPAR-gamma and AMPK Pathways.** *J Agric Food Chem* 2011.

58. Katayama K, Furuki R, Yokoyama H, Kaneko M, Tachibana M, Yoshida I, Nagase H, Tanaka K, Sakurai F, Mizuguchi H, et al: **Enhanced in vivo gene transfer into the placenta using RGD fiber-mutant adenovirus vector.** *Biomaterials* 2011.

59. Adekar SP, Segan AT, Chen C, Bermudez R, Elias MD, Selling BH, Kapadnis BP, Simpson LL, Simon PM, Dessain SK: **Enhanced neutralization potency of botulinum neurotoxin antibodies using a red blood cell-targeting fusion protein.** *Plos One* 2011, **6:**e17491.

60. Das DK, Mukherjee S, Ray D: **Erratum to: Resveratrol and red wine, healthy heart and longevity.** *Heart Fail Rev* 2011.

61. Hailei W, Zhifang R, Ping L, Yanchang G, Guosheng L, Jianming Y: **Improvement of the production of a red pigment in Penicillium sp. HSD07B synthesized during co-culture with Candida tropicalis.** *Bioresour Technol* 2011.

62. Seo I, Kim SH, Lee JE, Jeong SJ, Kim YC, Ahn KS, Lu J: **Ka-mi-kae-kyuk-tang oriental herbal cocktail attenuates cyclophosphamide-induced leukopenia side effects in mouse.** *Immunopharmacol Immunotoxicol* 2011.

63. Desmarets M, Noizat-Pirenne F: **[Murine models in blood transfusion: Allo-immunization, hemolysis.].** *Transfus Clin Biol* 2011.

64. Revuelta-Cervantes J, Mayoral R, Miranda S, Gonzalez-Rodriguez A, Fernandez M, Martin-Sanz P, Valverde AM: **Protein Tyrosine Phosphatase 1B (PTP1B) Deficiency Accelerates Hepatic Regeneration in Mice.** *Am J Pathol* 2011.

65. Andrikopoulos P, Fraser SP, Patterson L, Ahmad Z, Burcu H, Ottaviani D, Diss JK, Box C, Eccles SA, Djamgoz MB: **Angiogenic functions of voltage-gated Na+ channels in human endothelial cells: modulation of Vascular endothelial growth factor (VEGF) signalling.** *J Biol Chem* 2011.

66. Dautova Y, Zhang Y, Grace AA, Huang CL: **Atrial arrhythmogenic properties in wild-type and Scn5a+/- murine hearts.** *Exp Physiol* 2010, **95:**994-1007.

67. Guzadhur L, Pearcey SM, Duehmke RM, Jeevaratnam K, Hohmann AF, Zhang Y, Grace AA, Lei M, Huang CL: **Atrial arrhythmogenicity in aged Scn5a+/DeltaKPQ mice modeling long QT type 3 syndrome and its relationship to Na+ channel expression and cardiac conduction.** *Pflugers Arch* 2010, **460:**593-601.

68. Aanhaanen WT, Boukens BJ, Sizarov A, Wakker V, de Gier-de Vries C, van Ginneken AC, Moorman AF, Coronel R, Christoffels VM: **Defective Tbx2-dependent patterning of the atrioventricular canal myocardium causes accessory pathway formation in mice.** *J Clin Invest* 2011, **121:**534-544.

69. Martin CA, Zhang Y, Grace AA, Huang CL: **In vivo studies of Scn5a+/- mice modeling Brugada syndrome demonstrate both conduction and repolarization abnormalities.** *J Electrocardiol* 2010, **43:**433-439.

70. Martin CA, Zhang Y, Grace AA, Huang CL: **Increased right ventricular repolarization gradients promote arrhythmogenesis in a murine model of Brugada syndrome.** *J Cardiovasc Electrophysiol* 2010, **21:**1153-1159.

71. Martin CA, Guzadhur L, Grace AA, Lei M, Huang CL: **Mapping of reentrant spontaneous polymorphic ventricular tachycardia in a Scn5a+/- mouse model.** *Am J Physiol Heart Circ Physiol* 2011.

72. Albesa M, Ogrodnik J, Rougier JS, Abriel H: **Regulation of the cardiac sodium channel Nav1.5 by utrophin in dystrophin-deficient mice.** *Cardiovasc Res* 2011, **89:**320-328.

73. Lang F, Strutz-Seebohm N, Seebohm G, Lang UE: **Significance of SGK1 in the regulation of neuronal function.** *J Physiol* 2010, **588:**3349-3354.

74. Martin CA, Grace AA, Huang CL: **Spatial and temporal heterogeneities are localized to the right ventricular outflow tract in a heterozygotic Scn5a mouse model.** *Am J Physiol Heart Circ Physiol* 2011, **300:**H605-616.

75. Zhou C, Chen H, King JA, Sellak H, Kuebler WM, Yin J, Townsley MI, Shin HS, Wu S: **Alpha1G T-type calcium channel selectively regulates P-selectin surface expression in pulmonary capillary endothelium.** *Am J Physiol Lung Cell Mol Physiol* 2010, **299:**L86-97.

76. Wu S, Jian MY, Xu YC, Zhou C, Al-Mehdi AB, Liedtke W, Shin HS, Townsley MI: **Ca2+ entry via alpha1G and TRPV4 channels differentially regulates surface expression of P-selectin and barrier integrity in pulmonary capillary endothelium.** *Am J Physiol Lung Cell Mol Physiol* 2009, **297:**L650-657.

77. Park YG, Park HY, Lee CJ, Choi S, Jo S, Choi H, Kim YH, Shin HS, Llinas RR, Kim D: **Ca(V)3.1 is a tremor rhythm pacemaker in the inferior olive.** *Proc Natl Acad Sci U S A* 2010, **107:**10731-10736.

78. Ernst WL, Noebels JL: **Expanded alternative splice isoform profiling of the mouse Cav3.1/alpha1G T-type calcium channel.** *BMC Mol Biol* 2009, **10:**53.

79. Ernst WL, Zhang Y, Yoo JW, Ernst SJ, Noebels JL: **Genetic enhancement of thalamocortical network activity by elevating alpha 1g-mediated low-voltage-activated calcium current induces pure absence epilepsy.** *J Neurosci* 2009, **29:**1615-1625.

80. Choi S, Yu E, Kim D, Urbano FJ, Makarenko V, Shin HS, Llinas RR: **Subthreshold membrane potential oscillations in inferior olive neurons are dynamically regulated by P/Q- and T-type calcium channels: a study in mutant mice.** *J Physiol* 2010, **588:**3031-3043.

81. Park C, Kim JH, Yoon BE, Choi EJ, Lee CJ, Shin HS: **T-type channels control the opioidergic descending analgesia at the low threshold-spiking GABAergic neurons in the periaqueductal gray.** *Proc Natl Acad Sci U S A* 2010, **107:**14857-14862.

82. Zhang C, Bosch MA, Rick EA, Kelly MJ, Ronnekleiv OK: **17Beta-estradiol regulation of T-type calcium channels in gonadotropin-releasing hormone neurons.** *J Neurosci* 2009, **29:**10552-10562.

83. Gibbons SJ, Strege PR, Lei S, Roeder JL, Mazzone A, Ou Y, Rich A, Farrugia G: **The alpha1H Ca2+ channel subunit is expressed in mouse jejunal interstitial cells of Cajal and myocytes.** *J Cell Mol Med* 2009, **13:**4422-4431.

84. Markandeya YS, Fahey JM, Pluteanu F, Cribbs LL, Balijepalli RC: **Caveolin-3 regulates protein kinase A modulation of the Ca(V)3.2 (alpha1H) T-type Ca2+ channels.** *J Biol Chem* 2011, **286:**2433-2444.

85. Mizuta E, Shirai M, Arakawa K, Hidaka K, Miake J, Ninomiya H, Kato M, Shigemasa C, Shirayoshi Y, Hisatome I, Morisaki T: **Different distribution of Cav3.2 and Cav3.1 transcripts encoding T-type Ca(2+) channels in the embryonic heart of mice.** *Biomed Res* 2010, **31:**301-305.

86. Orestes P, Bojadzic D, Lee J, Leach E, Salajegheh R, Digruccio MR, Nelson MT, Todorovic SM: **Free radical signalling underlies inhibition of CaV3.2 T-type calcium channels by nitrous oxide in the pain pathway.** *J Physiol* 2011, **589:**135-148.

87. Rowell JJ, Mallik AK, Dugas-Ford J, Ragsdale CW: **Molecular analysis of neocortical layer structure in the ferret.** *J Comp Neurol* 2010, **518:**3272-3289.

88. Lee WY, Orestes P, Latham J, Naik AK, Nelson MT, Vitko I, Perez-Reyes E, Jevtovic-Todorovic V, Todorovic SM: **Molecular mechanisms of lipoic acid modulation of T-type calcium channels in pain pathway.** *J Neurosci* 2009, **29:**9500-9509.

89. Wildburger NC, Lin-Ye A, Baird MA, Lei D, Bao J: **Neuroprotective effects of blockers for T-type calcium channels.** *Mol Neurodegener* 2009, **4:**44.

90. Zamponi GW, Lory P, Perez-Reyes E: **Role of voltage-gated calcium channels in epilepsy.** *Pflugers Arch* 2010, **460:**395-403.

91. Barbara G, Alloui A, Nargeot J, Lory P, Eschalier A, Bourinet E, Chemin J: **T-type calcium channel inhibition underlies the analgesic effects of the endogenous lipoamino acids.** *J Neurosci* 2009, **29:**13106-13114.

92. Meissner M, Weissgerber P, Camacho Londono JE, Prenen J, Link S, Ruppenthal S, Molkentin JD, Lipp P, Nilius B, Freichel M, Flockerzi V: **Moderate calcium channel dysfunction in adult mice with inducible cardiomyocyte-specific excision of the CACNB2 gene.** *J Biol Chem* 2011.

93. Tang ZZ, Sharma S, Zheng S, Chawla G, Nikolic J, Black DL: **Regulation of the mutually exclusive exons 8a and 8 in the CaV1.2 calcium channel transcript by polypyrimidine tract binding protein.** *J Biol Chem* 2011.

94. Best JM, Foell JD, Buss CR, Delisle BP, Balijepalli RC, January CT, Kamp TJ: **The Small GTPase Rab11b Regulates Degradation of Surface Membrane L-Type Cav1.2 Channels.** *Am J Physiol Cell Physiol* 2011.

95. Asemu G, Fishbein K, Lao QZ, Ravindran A, Herbert R, Canuto HC, Spencer RG, Soldatov NM: **Cardiac phenotype induced by a dysfunctional alpha1C transgene: A general problem for the transgenic approach.** *Channels (Austin)* 2011, **5**.

96. Marshall MR, Clark JP, Westenbroek R, Yu FH, Scheuer T, Catterall WA: **Functional roles of a C-terminal signaling complex of CAV1 channels and A kinase anchoring protein-15 in brain neurons.** *J Biol Chem* 2011.

97. Fu Y, Westenbroek RE, Yu FH, Clark JP, 3rd, Marshall MR, Scheuer T, Catterall WA: **Deletion of the distal C-terminus of Cav1.2 channel leads to loss of Beta-adrenergic regulation and heart failure in vivo.** *J Biol Chem* 2011.

98. Chen X, Nakayama H, Zhang X, Ai X, Harris DM, Tang M, Zhang H, Szeto C, Stockbower K, Berretta RM, et al: **Calcium influx through Cav1.2 is a proximal signal for pathological cardiomyocyte hypertrophy.** *J Mol Cell Cardiol* 2011, **50:**460-470.

99. Lu Y, Zhang Y, Wang N, Pan Z, Gao X, Zhang F, Shan H, Luo X, Bai Y, Sun L, et al: **MicroRNA-328 contributes to adverse electrical remodeling in atrial fibrillation.** *Circulation* 2010, **122:**2378-2387.

100. Chang CC, Cao S, Kang S, Kai L, Tian X, Pandey P, Dunne SF, Luan CH, Surmeier DJ, Silverman RB: **Antagonism of 4-substituted 1,4-dihydropyridine-3,5-dicarboxylates toward voltage-dependent L-type Ca2+ channels Ca V 1.3 and Ca V 1.2.** *Bioorg Med Chem* 2010, **18:**3147-3158.

101. Kersten FF, van Wijk E, van Reeuwijk J, van der Zwaag B, Marker T, Peters TA, Katsanis N, Wolfrum U, Keunen JE, Roepman R, Kremer H: **Association of whirlin with Cav1.3 (alpha1D) channels in photoreceptors, defining a novel member of the usher protein network.** *Invest Ophthalmol Vis Sci* 2010, **51:**2338-2346.

102. Jenkins MA, Christel CJ, Jiao Y, Abiria S, Kim KY, Usachev YM, Obermair GJ, Colbran RJ, Lee A: **Ca2+-dependent facilitation of Cav1.3 Ca2+ channels by densin and Ca2+/calmodulin-dependent protein kinase II.** *J Neurosci* 2010, **30:**5125-5135.

103. Vandael DH, Marcantoni A, Mahapatra S, Caro A, Ruth P, Zuccotti A, Knipper M, Carbone E: **Ca(v)1.3 and BK channels for timing and regulating cell firing.** *Mol Neurobiol* 2010, **42:**185-198.

104. Meehan CF, Sukiasyan N, Zhang M, Nielsen JB, Hultborn H: **Intrinsic properties of mouse lumbar motoneurons revealed by intracellular recording in vivo.** *J Neurophysiol* 2010, **103:**2599-2610.

105. Baig SM, Koschak A, Lieb A, Gebhart M, Dafinger C, Nurnberg G, Ali A, Ahmad I, Sinnegger-Brauns MJ, Brandt N, et al: **Loss of Ca(v)1.3 (CACNA1D) function in a human channelopathy with bradycardia and congenital deafness.** *Nat Neurosci* 2011, **14:**77-84.

106. Marcantoni A, Vandael DH, Mahapatra S, Carabelli V, Sinnegger-Brauns MJ, Striessnig J, Carbone E: **Loss of Cav1.3 channels reveals the critical role of L-type and BK channel coupling in pacemaking mouse adrenal chromaffin cells.** *J Neurosci* 2010, **30:**491-504.

107. Kurokawa K, Mizuno K, Shibasaki M, Ohkuma S: **Regulation of ryanodine receptors by dopamine D1 receptors during methamphetamine-induced place conditioning.** *J Neurochem* 2010, **115:**1206-1214.

108. Li J, Zhao L, Ferries IK, Jiang L, Desta MZ, Yu X, Yang Z, Duncan RL, Turner CH: **Skeletal phenotype of mice with a null mutation in Cav 1.3 L-type calcium channel.** *J Musculoskelet Neuronal Interact* 2010, **10:**180-187.

109. Shibasaki M, Kurokawa K, Ohkuma S: **Upregulation of L-type Ca(v)1 channels in the development of psychological dependence.** *Synapse* 2010, **64:**440-444.

110. Adams PJ, Snutch TP: **Calcium channelopathies: voltage-gated calcium channels.** *Subcell Biochem* 2007, **45:**215-251.

111. Griessmeier K, Cuny H, Rotzer K, Griesbeck O, Harz H, Biel M, Wahl-Schott C: **Calmodulin is a functional regulator of Cav1.4 L-type Ca2+ channels.** *J Biol Chem* 2009, **284:**29809-29816.

112. Striessnig J, Bolz HJ, Koschak A: **Channelopathies in Cav1.1, Cav1.3, and Cav1.4 voltage-gated L-type Ca2+ channels.** *Pflugers Arch* 2010, **460:**361-374.

113. Lodha N, Bonfield S, Orton NC, Doering CJ, McRory JE, Mema SC, Rehak R, Sauve Y, Tobias R, Stell WK, Bech-Hansen NT: **Congenital stationary night blindness in mice - a tale of two cacna1f mutants.** *Adv Exp Med Biol* 2010, **664:**549-558.

114. Jha MK, Badou A, Meissner M, McRory JE, Freichel M, Flockerzi V, Flavell RA: **Defective survival of naive CD8+ T lymphocytes in the absence of the beta3 regulatory subunit of voltage-gated calcium channels.** *Nat Immunol* 2009, **10:**1275-1282.

115. Lorenzon NM, Beam KG: **Disease causing mutations of calcium channels.** *Channels (Austin)* 2008, **2:**163-179.

116. Raven MA, Orton NC, Nassar H, Williams GA, Stell WK, Jacobs GH, Bech-Hansen NT, Reese BE: **Early afferent signaling in the outer plexiform layer regulates development of horizontal cell morphology.** *J Comp Neurol* 2008, **506:**745-758.

117. Specht D, Wu SB, Turner P, Dearden P, Koentgen F, Wolfrum U, Maw M, Brandstatter JH, tom Dieck S: **Effects of presynaptic mutations on a postsynaptic Cacna1s calcium channel colocalized with mGluR6 at mouse photoreceptor ribbon synapses.** *Invest Ophthalmol Vis Sci* 2009, **50:**505-515.

118. Doering CJ, Rehak R, Bonfield S, Peloquin JB, Stell WK, Mema SC, Sauve Y, McRory JE: **Modified Ca(v)1.4 expression in the Cacna1f(nob2) mouse due to alternative splicing of an ETn inserted in exon 2.** *Plos One* 2008, **3:**e2538.

119. Baehr W, Frederick JM: **Naturally occurring animal models with outer retina phenotypes.** *Vision Res* 2009, **49:**2636-2652.

120. Tuluc P, Molenda N, Schlick B, Obermair GJ, Flucher BE, Jurkat-Rott K: **A CaV1.1 Ca2+ channel splice variant with high conductance and voltage-sensitivity alters EC coupling in developing skeletal muscle.** *Biophys J* 2009, **96:**35-44.

121. Pietri-Rouxel F, Gentil C, Vassilopoulos S, Baas D, Mouisel E, Ferry A, Vignaud A, Hourde C, Marty I, Schaeffer L, et al: **DHPR alpha1S subunit controls skeletal muscle mass and morphogenesis.** *EMBO J* 2010, **29:**643-654.

122. Pirone A, Schredelseker J, Tuluc P, Gravino E, Fortunato G, Flucher BE, Carsana A, Salvatore F, Grabner M: **Identification and functional characterization of malignant hyperthermia mutation T1354S in the outer pore of the Cavalpha1S-subunit.** *Am J Physiol Cell Physiol* 2010, **299:**C1345-1354.

123. Maclennan DH, Zvaritch E: **Mechanistic models for muscle diseases and disorders originating in the sarcoplasmic reticulum.** *Biochim Biophys Acta* 2010.

124. Duchesne A, Eggen A: **Radiation hybrid mapping of genes and newly identified microsatellites in candidate regions for bovine arthrogryposis-palatoschisis and progressive ataxia based on comparative data from man, mouse and rat.** *J Anim Breed Genet* 2005, **122 Suppl 1:**28-35.

125. Strube C: **Absence of regulation of the T-type calcium current by Cav1.1, beta1a and gamma1 dihydropyridine receptor subunits in skeletal muscle cells.** *Pflugers Arch* 2008, **455:**921-927.

126. Yamazaki K, Shigetomi E, Ikeda R, Nishida M, Kiyonaka S, Mori Y, Kato F: **Blocker-resistant presynaptic voltage-dependent Ca2+ channels underlying glutamate release in mice nucleus tractus solitarii.** *Brain Res* 2006, **1104:**103-113.

127. Takahashi E, Nagasu T: **Enhanced expression of Ca2+ channel alpha1A and beta4 subunits and phosphorylated tyrosine hydroxylase in the adrenal gland of N-type Ca2+ channel alpha1B subunit-deficient mice with a CBA/JN genetic background.** *Comp Med* 2006, **56:**168-175.

128. Katoh M: **Identification and characterization of human ARHGAP23 gene in silico.** *Int J Oncol* 2004, **25:**535-540.

129. Taylor JR, Zheng Z, Wang ZM, Payne AM, Messi ML, Delbono O: **Increased CaVbeta1A expression with aging contributes to skeletal muscle weakness.** *Aging Cell* 2009, **8:**584-594.

130. Leuranguer V, Papadopoulos S, Beam KG: **Organization of calcium channel beta1a subunits in triad junctions in skeletal muscle.** *J Biol Chem* 2006, **281:**3521-3527.

131. Garcia MC, Carrillo E, Galindo JM, Hernandez A, Copello JA, Fill M, Sanchez JA: **Short-term regulation of excitation-contraction coupling by the beta1a subunit in adult mouse skeletal muscle.** *Biophys J* 2005, **89:**3976-3984.

132. Murakami M, Miyoshi I, Suzuki T, Sasano H, Iijima T: **Structures of the murine genes for the beta1- and beta4-subunits of the voltage-dependent calcium channel.** *J Mol Neurosci* 2003, **21:**13-21.

133. Xie D, Hu P, Xiao Z, Wu W, Chen Y, Xia K: **Subunits of voltage-gated calcium channels in murine spiral ganglion cells.** *Acta Otolaryngol* 2007, **127:**8-12.

134. Finlin BS, Correll RN, Pang C, Crump SM, Satin J, Andres DA: **Analysis of the complex between Ca2+ channel beta-subunit and the Rem GTPase.** *J Biol Chem* 2006, **281:**23557-23566.

135. Held B, Tsvilovskyy V, Meissner M, Kaestner L, Ludwig A, Mossmang S, Lipp P, Freichel M, Flockerzi V: **Ca2+ channel currents and contraction in CaVbeta3-deficient ileum smooth muscle from mouse.** *Cell Calcium* 2007, **42:**477-487.

136. Neef J, Gehrt A, Bulankina AV, Meyer AC, Riedel D, Gregg RG, Strenzke N, Moser T: **The Ca2+ channel subunit beta2 regulates Ca2+ channel abundance and function in inner hair cells and is required for hearing.** *J Neurosci* 2009, **29:**10730-10740.

137. Ebert AM, McAnelly CA, Srinivasan A, Mueller RL, Garrity DB, Garrity DM: **The calcium channel beta2 (CACNB2) subunit repertoire in teleosts.** *BMC Mol Biol* 2008, **9:**38.

138. Murakami M, Ohba T, Takahashi Y, Watanabe H, Miyoshi I, Nakayama S, Ono K, Ito H, Iijima T: **Identification of a cardiac isoform of the murine calcium channel alpha1C (Cav1.2-a) subunit and its preferential binding with the beta2 subunit.** *J Mol Cell Cardiol* 2006, **41:**115-125.

139. Amaral AG, Rafacho A, Machado de Oliveira CA, Batista TM, Ribeiro RA, Latorraca MQ, Boschero AC, Carneiro EM: **Leucine supplementation augments insulin secretion in pancreatic islets of malnourished mice.** *Pancreas* 2010, **39:**847-855.

140. Grueter CE, Abiria SA, Dzhura I, Wu Y, Ham AJ, Mohler PJ, Anderson ME, Colbran RJ: **L-type Ca2+ channel facilitation mediated by phosphorylation of the beta subunit by CaMKII.** *Mol Cell* 2006, **23:**641-650.

141. Kaja S, Todorov B, van de Ven RC, Ferrari MD, Frants RR, van den Maagdenberg AM, Plomp JJ: **Redundancy of Cav2.1 channel accessory subunits in transmitter release at the mouse neuromuscular junction.** *Brain Res* 2007, **1143:**92-101.

142. Thomsen MB, Foster E, Nguyen KH, Sosunov EA: **Transcriptional and electrophysiological consequences of KChIP2-mediated regulation of CaV1.2.** *Channels (Austin)* 2009, **3:**308-310.

143. Kuhn S, Knirsch M, Ruttiger L, Kasperek S, Winter H, Freichel M, Flockerzi V, Knipper M, Engel J: **Ba2+ currents in inner and outer hair cells of mice lacking the voltage-dependent Ca2+ channel subunits beta3 or beta4.** *Channels (Austin)* 2009, **3:**366-376.

144. Ohta T, Ohba T, Suzuki T, Watanabe H, Sasano H, Murakami M: **Decreased calcium channel currents and facilitated epinephrine release in the Ca2+ channel beta3 subunit-null mice.** *Biochem Biophys Res Commun* 2010, **394:**464-469.

145. Bros M, Dexheimer N, Ross R, Trojandt S, Hohn Y, Tampe J, Sutter A, Jahrling F, Grabbe S, Reske-Kunz AB: **Differential gene expression analysis identifies murine Cacnb3 as strongly upregulated in distinct dendritic cell populations upon stimulation.** *Gene* 2011, **472:**18-27.

146. Frigeri A, Iacobas DA, Iacobas S, Nicchia GP, Desaphy JF, Camerino DC, Svelto M, Spray DC: **Effect of microgravity on gene expression in mouse brain.** *Exp Brain Res* 2008, **191:**289-300.

147. Bernardo JF, Magyar CE, Sneddon WB, Friedman PA: **Impaired renal calcium absorption in mice lacking calcium channel beta 3 subunits.** *Can J Physiol Pharmacol* 2009, **87:**522-530.

148. Murakami M, Nakagawasai O, Yanai K, Nunoki K, Tan-No K, Tadano T, Iijima T: **Modified behavioral characteristics following ablation of the voltage-dependent calcium channel beta3 subunit.** *Brain Res* 2007, **1160:**102-112.

149. Murakami M, Ohba T, Xu F, Satoh E, Miyoshi I, Suzuki T, Takahashi Y, Takahashi E, Watanabe H, Ono K, et al: **Modified sympathetic nerve system activity with overexpression of the voltage-dependent calcium channel beta3 subunit.** *J Biol Chem* 2008, **283:**24554-24560.

150. Beguin P, Ng YJ, Krause C, Mahalakshmi RN, Ng MY, Hunziker W: **RGK small GTP-binding proteins interact with the nucleotide kinase domain of Ca2+-channel beta-subunits via an uncommon effector binding domain.** *J Biol Chem* 2007, **282:**11509-11520.

151. Subramanyam P, Obermair GJ, Baumgartner S, Gebhart M, Striessnig J, Kaufmann WA, Geley S, Flucher BE: **Activity and calcium regulate nuclear targeting of the calcium channel beta4b subunit in nerve and muscle cells.** *Channels (Austin)* 2009, **3:**343-355.

152. Murphree LJ, Rundhaugen LM, Kelly KM: **Animal models of geriatric epilepsy.** *Int Rev Neurobiol* 2007, **81:**29-40.

153. Yamakawa K: **[Development of the molecular genetics of epilepsy].** *No To Shinkei* 2005, **57:**181-194.

154. Reichhart N, Milenkovic VM, Halsband CA, Cordeiro S, Strauss O: **Effect of bestrophin-1 on L-type Ca2+ channel activity depends on the Ca2+ channel beta-subunit.** *Exp Eye Res* 2010, **91:**630-639.

155. Takahashi E, Nagasu T: **Expression pattern of voltage-dependent calcium channel alpha1 and beta subunits in adrenal gland of N-type Ca2+ channel alpha1B subunit gene-deficient mice.** *Mol Cell Biochem* 2005, **271:**91-99.

156. Abouda H, Hizem Y, Gargouri A, Depienne C, Bouteiller D, Riant F, Tournier-Lasserve E, Gourfinkel-An I, LeGuern E, Gouider R: **Familial form of typical childhood absence epilepsy in a consanguineous context.** *Epilepsia* 2010, **51:**1889-1893.

157. Leikauf GD, Concel VJ, Liu P, Bein K, Berndt A, Ganguly K, Jang AS, Brant KA, Dietsch M, Pope-Varsalona H, et al: **Haplotype Association Mapping of Acute Lung Injury in Mice Implicates Activin A Receptor, Type 1.** *Am J Respir Crit Care Med* 2011.

158. Devanagondi R, Egami K, LeDoux MS, Hess EJ, Jinnah HA: **Neuroanatomical substrates for paroxysmal dyskinesia in lethargic mice.** *Neurobiol Dis* 2007, **27:**249-257.

159. Silveira LF, Teles MG, Trarbach EB, Latronico AC: **Role of kisspeptin/GPR54 system in human reproductive axis.** *Front Horm Res* 2010, **39:**13-24.

160. Tseng CJ, Lin YH, Han CP: **BD Pharmingen purified mouse anti-cytokeratin 7 monoclonal antibody (clone RCK105) should not be mistaken for BD Biosciences anti-cytokeratin CAM5.2 reagent (clone CAM 5.2).** *Am J Obstet Gynecol* 2011, **204:**e14.

161. Sei Y, Lu X, Liou A, Zhao X, Wank SA: **A stem cell marker-expressing subset of enteroendocrine cells resides at the crypt base in the small intestine.** *Am J Physiol Gastrointest Liver Physiol* 2011, **300:**G345-356.

162. Sarkar S, Swiercz R, Kantara C, Hajjar KA, Singh P: **Annexin A2 mediates up-regulation of NF-kappaB, beta-catenin, and stem cell in response to progastrin in mice and HEK-293 cells.** *Gastroenterology* 2011, **140:**583-595 e584.

163. Griffiths DS, Li J, Dawson MA, Trotter MW, Cheng YH, Smith AM, Mansfield W, Liu P, Kouzarides T, Nichols J, et al: **LIF-independent JAK signalling to chromatin in embryonic stem cells uncovered from an adult stem cell disease.** *Nat Cell Biol* 2011, **13:**13-21.

164. Degrelle SA, Le Cao KA, Heyman Y, Everts RE, Campion E, Richard C, Ducroix-Crepy C, Tian XC, Lewin HA, Renard JP, et al: **A small set of extra-embryonic genes defines a new landmark for bovine embryo staging.** *Reproduction* 2011, **141:**79-89.

165. Chen MM, Lee CY, Leland HA, Silletti S: **Modification of the L1-CAM carboxy-terminus in pancreatic adenocarcinoma cells.** *Tumour Biol* 2011, **32:**347-357.

166. Burren OS, Adlem EC, Achuthan P, Christensen M, Coulson RM, Todd JA: **T1DBase: update 2011, organization and presentation of large-scale data sets for type 1 diabetes research.** *Nucleic Acids Res* 2011, **39:**D997-1001.

167. Zernicka-Goetz M, Huang S: **Stochasticity versus determinism in development: a false dichotomy?** *Nat Rev Genet* 2010, **11:**743-744.

168. Ying Y, Xingfen Y, Wengai Z, Jinheng C, Jinyu X, Guangyu Y, Xiaohua T, Xiaoping X, Xikun X, Junming H, Xiang G: **Combined in vitro tests as an alternative to in vivo eye irritation tests.** *Altern Lab Anim* 2010, **38:**303-314.

169. Xu Y, Zhao H, Zheng Y, Gu Q, Ma J, Xu X: **A novel antiangiogenic peptide derived from hepatocyte growth factor inhibits neovascularization in vitro and in vivo.** *Mol Vis* 2010, **16:**1982-1995.

170. Yuan Z, Zou F, Liu Y: **Bayesian Multiple Quantitative Trait Loci Mapping for Recombinant Inbred Intercrosses.** *Genetics* 2011.

171. Wang MY, Hurn J, Peng L, Nowicki D, Anderson G: **A multigeneration reproductive and developmental safety evaluation of authentic Morinda citrifolia (noni) juice.** *J Toxicol Sci* 2011, **36:**81-85.

172. Thoss M, Ilmonen P, Musolf K, Penn DJ: **Major histocompatibility complex heterozygosity enhances reproductive success.** *Mol Ecol* 2011.

173. Tam JC, Lau KM, Liu CL, To MH, Kwok HF, Lai KK, Lau CP, Ko CH, Leung PC, Fung KP, Lau CB: **The in vivo and in vitro diabetic wound healing effects of a 2-herb formula and its mechanisms of action.** *J Ethnopharmacol* 2011.

174. Stouder C, Somm E, Paoloni-Giacobino A: **Prenatal exposure to ethanol: A specific effect on the H19 gene in sperm.** *Reprod Toxicol* 2011.

175. Snegovskikh VV, Bhandari V, Wright JR, Tadesse S, Morgan T, Macneill C, Foyouzi N, Park JS, Wang Y, Norwitz ER: **Surfactant Protein-A (SP-A) Selectively Inhibits Prostaglandin F2{alpha} (PGF2{alpha}) Production in Term Decidua: Implications for the Onset of Labor.** *J Clin Endocrinol Metab* 2011.

176. Smith AM, Adler FR, McAuley JL, Gutenkunst RN, Ribeiro RM, McCullers JA, Perelson AS: **Effect of 1918 PB1-F2 Expression on Influenza A Virus Infection Kinetics.** *Plos Comput Biol* 2011, **7:**e1001081.

177. Quan L, Stassen AP, Ruivenkamp CA, van Wezel T, Fijneman RJ, Hutson A, Kakarlapudi N, Hart AA, Demant P: **Most lung and colon cancer susceptibility genes are pair-wise linked in mice, humans and rats.** *Plos One* 2011, **6:**e14727.

178. Ozawa M, Basnet S, Burley LM, Neumann G, Hatta M, Kawaoka Y: **Impact of amino acid mutations in PB2, PB1-F2, and NS1 on the replication and pathogenicity of pandemic (H1N1) 2009 influenza viruses.** *J Virol* 2011.

179. Jiao Y, Jiao F, Yan J, Xiong Q, Shriner D, Hasty K, Stuart J, Gu W: **Identifying a major locus that regulates spontaneous arthritis in IL-1ra-deficient mice and analysis of potential candidates.** *Genet Res (Camb)* 2011**:**1-9.
